# Bioactive Compounds from *Polygala tenuifolia* and Their Inhibitory Effects on Lipopolysaccharide-Stimulated Pro-inflammatory Cytokine Production in Bone Marrow-Derived Dendritic Cells

**DOI:** 10.3390/plants9091240

**Published:** 2020-09-20

**Authors:** Le Ba Vinh, Myungsook Heo, Nguyen Viet Phong, Irshad Ali, Young Sang Koh, Young Ho Kim, Seo Young Yang

**Affiliations:** 1 College of Pharmacy, Chungnam National University, Daejeon 34134, Korea; vinhrooney@gmail.com (L.B.V.); inyl1110@naver.com (M.H.); 2 Institute of Marine Biochemistry (IMBC), Vietnam Academy of Science and Technology (VAST), Hanoi 100000, Vietnam; ngvietphong@gmail.com; 3 School of Medicine and Jeju Research Center for Natural Medicine, Jeju National University, Jeju 63243, Korea; irshad.qau200@gmail.com (I.A.); yskoh7@jejunu.ac.kr (Y.S.K.)

**Keywords:** *Polygala tenuifolia*, phenolic glycosides, saponins, anti-inflammatory effect, bioactive compound

## Abstract

The roots of *Polygala tenuifolia* Wild (Polygalaceae), which is among the most important components of traditional Chinese herbal medicine, have been widely used for over 1000 years to treat a variety of diseases. In the current investigation of secondary metabolites with anti-inflammatory properties from Korean medicinal plants, a phytochemical constituent study led to the isolation of 15 compounds (**1**–**15**) from the roots of *P. tenuifolia* via a combination of chromatographic methods. Their structures were determined by means of spectroscopic data such as nuclear magnetic resonance (NMR), 1D- and 2D-NMR, and liquid chromatography-mass spectrometry (LC-MS). As the obtained results, the isolated compounds were divided into two groups—phenolic glycosides (**1**–**9**) and triterpenoid saponins (**10**–**15**). The anti-inflammatory effects of crude extracts, fractions, and isolated compounds were investigated on the production of the pro-inflammatory cytokines interleukin (IL)-12 p40, IL-6, and tumour necrosis factor-α in lipopolysaccharide-stimulated bone marrow-derived dendritic cells. The IC_50_ values, ranging from 0.08 ± 0.01 to 21.05 ± 0.40 μM, indicated potent inhibitory effects of the isolated compounds on the production of all three pro-inflammatory cytokines. In particular, compounds **3**–**12**, **14**, and **15** showed promising anti-inflammatory activity. These results suggest that phenolic and triterpenoid saponins from *P. tenuifolia* may be excellent anti-inflammatory agents.

## 1. Introduction

Chronic inflammation, which is associated with complications such as osteoarthritis and cancer, is currently the most challenging public health issue. Inflammation is at the root of several non-communicable diseases, which kill approximately 40 million people worldwide each year and account for 70% of all deaths, according to the World Health Organization. Inflammation is a complex biological response to tissue injury that can be caused by mechanical stimulation, microbial invasion, and irritants [1,2]. The inflammatory process often involves the release of biochemical mediators and can lead to the development of diseases such as cancer, rheumatoid arthritis, osteoarthritis, and diabetes mellitus [3].

Inflammatory cells produce cytokines, including interleukin (IL)-12 p40, IL-6, and tumor necrosis factor (TNF)-α. IL-12 p40, IL-6, and TNF-α play important roles in the inflammatory response, as well as cell apoptosis and transformation [1]. Many studies have focused on the inhibition of pro-inflammatory cytokines in an effort to decrease inflammation-related diseases [4,5,6].

*Polygala tenuifolia* is a herb in the Polygalaceae family, which is widely distributed in Asia. In traditional folk medicine, *P. tenuifolia* has been used for thousands of years as an expectorant and stimulant to treat bronchial asthma, chronic bronchitis, and whooping cough [7]. The major components of *P. tenuifolia* include triterpenoid saponins [8], xanthone glycosides [9], phenolic glycosides [10], and oligosaccharide ester derivatives [11]. Isolated components from *P. tenuifolia* roots exhibit diverse pharmacological effects, including anti-inflammatory [7] and anti-diabetic properties. In particular, saponin compounds (onjisaponins A, B, E, F, and G) from the roots of *P. tenuifolia* have been used to treat psychosis, whereas xanthone constituents were found to inhibit neuraminidases from influenza A viruses [12].

Natural bioactive compounds, such as phenolics, triterpene saponins, and carbohydrates, are responsible for the pharmaceutical activities of medicinal herbs [13,14]. In an effort to discover new anti-inflammatory agents in medicinal plants, we isolated 15 compounds from a methanol (MeOH) extract of *P. tenuifolia* roots using bioassay-guided and chromatographic separation methods. In addition, the anti-inflammatory activities of crude MeOH extract, dichloromethane (DCM), ethyl acetate (EtOAc), and aqueous fractions, and of isolated compounds, were examined by measuring their inhibitory effects on lipopolysaccharide (LPS)-induced expression of the pro-inflammatory cytokines IL-12 p40, IL-6, and TNF-α in bone marrow-derived dendritic cells (BMDCs). These results suggest that *P. tenuifolia* extracts and isolated compounds **3**−**12**, **14**, and **15** have significant anti-inflammatory effects and may be candidates for the treatment of inflammation and other related diseases.

## 2. Results and Discussion 

**Figure 1:**
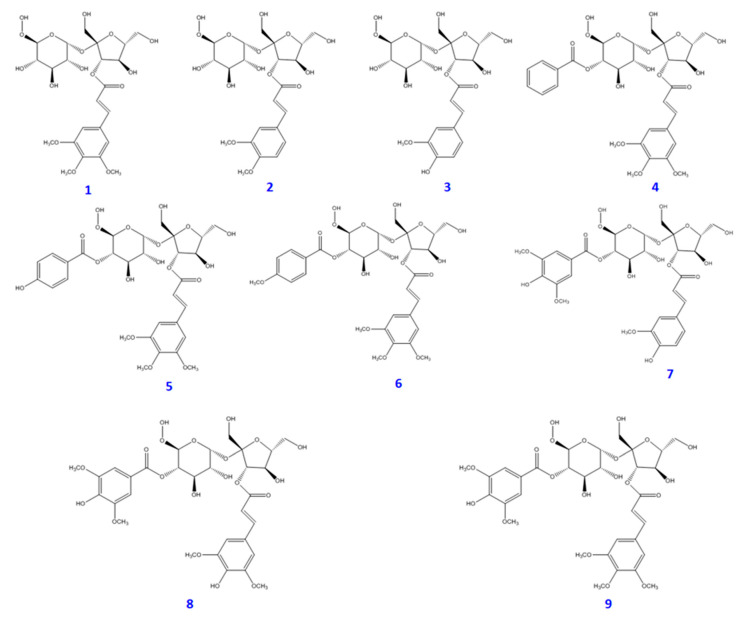
Chemical structures of phenolic constituents (**1**–**9**) from *Polygala tenuifolia* roots.

**Figure 2:**
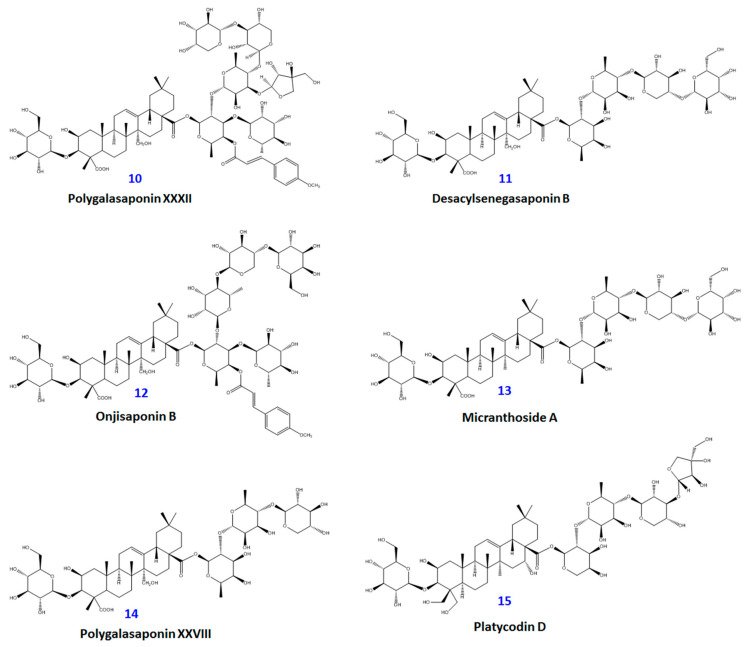
Chemical structures of triterpene saponin constituents (**10**–**15**) from *P. tenuifolia* roots.

Bioassay-guided fractionation of a MeOH extract of dried *P. tenuifolia* roots yielded 15 distinct compounds (**1**−**15**). These compounds, shown in Figure 1 and Figure 2, are as follows: glomeratose A (**1**), 3′-*O*-(*O*-methylferuloyl)sucrose (**2**), sibiricose A5 (**3**), 4-*O*-benzoyl-3′-*O*-(*O*-methylsinapoyl)sucrose (**4**), tenuifoliside A (**5**), 6-*O*-(*O*-methyl-*p*-benzoyl)-3′-*O*-(*O*-methylsinapoyl)sucrose (**6**), arillanin A (**7**), 6,3′-di-*O*-sinapoylsucrose (**8**), tenuifoliside C (**9**), polygalasaponin XXXII (**10**), desacylsenegasaponin B (**11**), onjisaponin B (**12**), micranthoside A (**13**), polygalasaponin XXVIII (**14**), and platycodin D (**15**). Their structures were characterized by nuclear magnetic resonance (NMR) spectroscopy (^1^H-NMR, ^13^C-NMR, COSY, HMQC, and HMBC spectra) and liquid chromatography-mass spectrometry (LC-MS) (see Supplementary Materials), and compared with structures reported in the literature. The detailed spectroscopic data of the isolated compounds were also reported. As the obtained results, the purified compounds were divided into two groups—phenolic glycosides (**1**-**9**) and triterpenoid saponins (**10**–**15**). Many of these phenolic glycosides and triterpenoid saponins are commonly found in *Polygala* species, such as *P. arillata* [15], *P. sibirica* [16], and *P. aureocauda* [17]. However, this is the first report of compound **15** being isolated from the genus *Polygala*.

**Figure 3:**
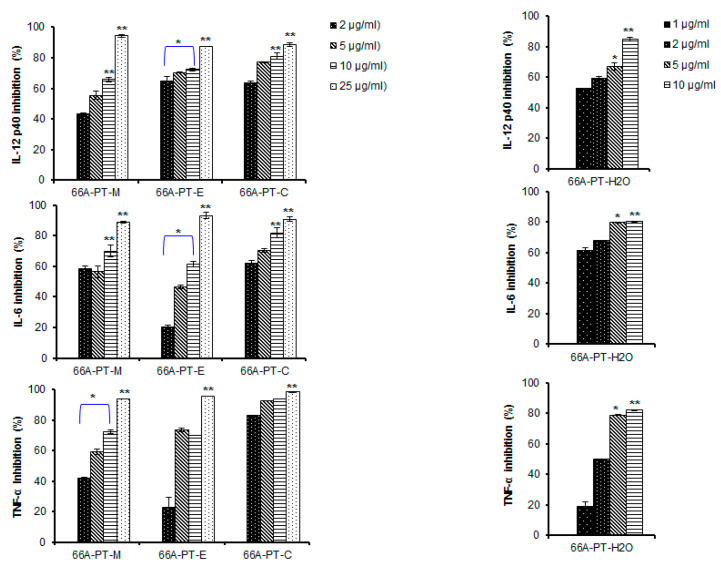
Effect of crude extracts (1, 2, 5, 10, and 15 µg/mL, respectively) on IL-12 p40, IL-6, and TNF-α production by LPS-stimulated bone marrow-derived dendritic cells (BMDCs). (* *P* < 0.05), (** *P* <0.01) versus compound untreated BMDCs in the presence of LPS. BMDCs were pre-treated with respective extracts at the indicated concentrations for 1 h and then stimulated with LPS (10 ng/mL) for 18 h. Inhibitory concentrations of IL-12 p40, IL-6, and TNF-α in the culture supernatant were determined by ELISA assay.

By studying how anti-inflammatory compounds inhibit the expression of inflammatory mediators such as IL-12 p40, IL-6, and TNF-α, therapeutic targets for anti-inflammatory agents can be identified for health promotion and disease prevention [6,18]. In an effort to discover new anti-inflammatory agents from medicinal plants, we extracted the roots of *P. tenuifolia* three times with MeOH. The crude extract was used to treat LPS-stimulated BMDCs and evaluate their production of cytokines. The MeOH extract of *P. tenuifolia* inhibited the production of IL-12 p40, IL-6, and TNF-α (IC_50_ = 3.38, 1.65, and 3.09 μg/mL, respectively) (Figure 3). Given the high anti-inflammatory activity of the MeOH extract, its components were further separated into DCM, EtOAc, and water fractions. As shown in Table 1, the water fraction strongly inhibited IL-12 p40, IL-6, and TNF-α production (IC_50_ = 0.94, 0.24, and 2.43 μg/mL, respectively). Therefore, the aqueous fraction was used to isolate individual active compounds. Using chromatographic techniques (silica gel column chromatography (CC); RP-C18 CC and Sephadex LH-20 columns), 15 compounds (**1**–**15**) were isolated from the water fraction of *P. tenuifolia* root extracts.

**Figure 4:**
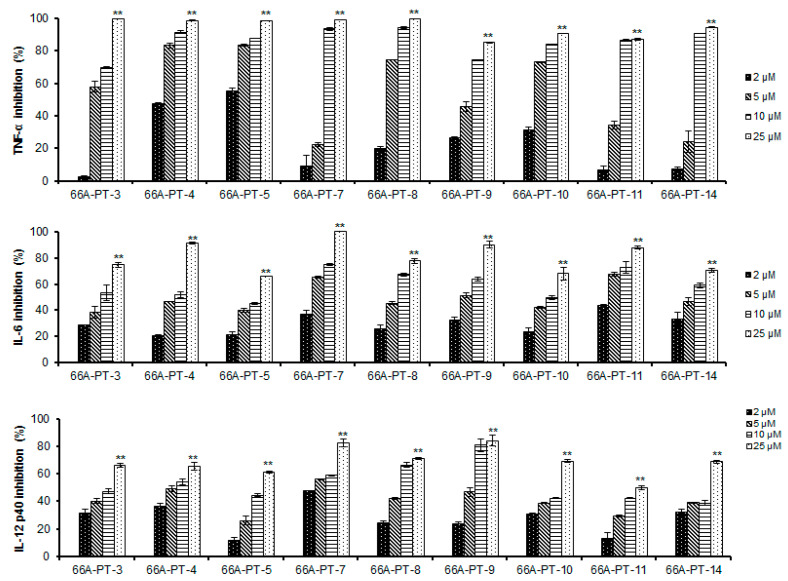
Effect of isolated compounds (**3**–**5**, **7**–**11**, and **14**) (2, 5, 10, and 15 µg/mL, respectively) on IL-12 p40, IL-6, and TNF-α production by LPS-stimulated BMDCs. BMDCs (bone marrow-derived dendritic cells) were pre-treated with respective compounds at the indicated concentrations for 1 h and then stimulated with LPS (10 ng/mL) for 18 h. Inhibitory concentrations of IL-12 p40, IL-6, and TNF-α in the culture supernatant were determined by ELISA assay. (* *P* < 0.05), (** *P* < 0.01) versus compound untreated BMDCs in the presence of LPS.

**Figure 5:**
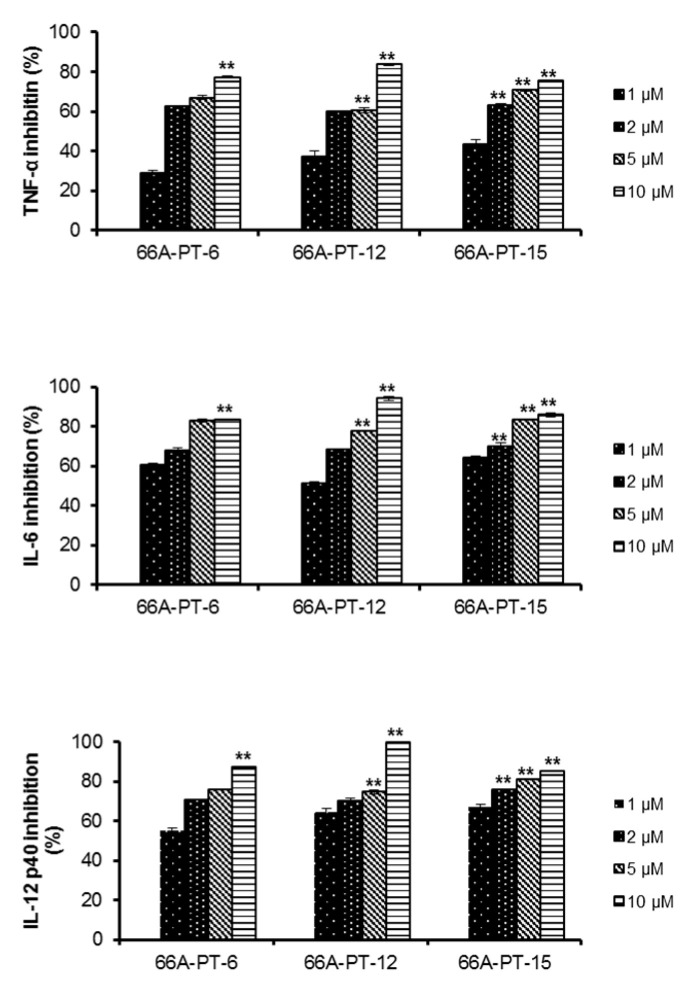
Effect of isolated compounds (**6**, **12**, and **15**) (1, 2, 5, and 10 µg/mL, respectively) on IL-12 p40, IL-6, and TNF-α production by LPS-stimulated BMDCs. BMDCs (bone marrow-derived dendritic cells) were pre-treated with respective compounds at the indicated concentrations for 1 h and then stimulated with LPS (10 ng/mL) for 18 h. Inhibitory concentrations of IL-12 p40, IL-6, and TNF-α in the culture supernatant were determined by ELISA assay. (* *P* < 0.05), (** *P* < 0.01) versus compound untreated BMDCs in the presence of LPS.

The isolated compounds were assessed in terms of their effects on the production of IL-12 p40. Compounds **3**–**10** and **12**–**15** greatly inhibited IL-12 p40 production, with IC_50_ values ranging from 0.08 ± 0.01 to 14.34 ± 0.03 µM, whereas compound **11** showed a moderate inhibitory effect on IL-12 p40 production (IC_50_ value of 21.05 ± 0.40) (Figure 4). Compounds **3**–**15** greatly inhibited IL-6 and TNF-α production, with IC_50_ values ranging from 0.24 ± 0.06 to 9.04 ± 0.05 and from 1.04 ± 0.12 to 6.34 ± 0.12 µM, respectively (Figure 5). The anti-inflammatory activities of the isolated compounds were comparable to those of the positive control, SB203580 (IC_50_ values of 5.00 ± 0.01, 3.50 ± 0.02, and 7.20 ± 0.02 µM, respectively) (Table 2). The extracts and isolated compounds were further assessed in terms of their effects on the viability of BMDCs using a colorimetric MTT (3-(4,5-dimethylthiazol-2-yl)-2,5-diphenyltetrazolium bromide) assay. Compounds **1**, **2**, and **13** showed strong cytotoxicity toward the BMDCs, whereas the other compounds displayed no notable cytotoxicity.

The structure–activity relationship (SAR) in phenolic glycosides and triterpenoid saponins may be deduced from anti-inflammatory effects. Consideration of the SAR of these triterpenoid saponins (**10**-**15**) suggests that the presence of the sugar units at C-3 and/or C-28 of the aglycon might play an important role in the anti-inflammatory inhibitory activity of these active compounds. In addition, the presence of substitute groups in molecules of phenolic glycosides (**1**–**9**), specifically –OH, –OCH_3_, and –COOH also clearly affect the IC_50_ values. Hence, further study is warranted to understand the SAR between the sugar moieties in aglycon and their pharmacological properties, particularly in vivo.

Natural products and related compounds are powerful alternatives for drug development. Phenolic and saponin constituents are important secondary metabolites in both medicinal plants and marine organisms that exhibit diverse pharmacological effects, such as anti-inflammatory [8], anti-cancer [19], anti-diabetic [20], anti-cardiovascular [21], and anti-oxidant activities [22]. Although there have been a few studies on the anti-inflammatory effects of *P. tenuifolia* [7], to the best of our knowledge, this is the first report on the inhibitory effects of individual constituents of *P. tenuifolia* roots on pro-inflammatory cytokine production in LPS-stimulated BMDCs.

In summary, 15 compounds (**1**–**15**) from *P. tenuifolia* roots were isolated via chromatographic separation techniques (silica gel CC with RP-C18 CC and Sephadex LH-20 columns). Their structures were unambiguously established by spectroscopic methods (1D-2D NMR and LC-MS), and their inhibitory effects on pro-inflammatory cytokine (IL-12 p40, IL-6, and TNF-α) production were characterized. The potential anti-inflammatory effects of the isolated compounds (**1**–**15**) increase our understanding of the chemotaxonomic properties of the Polygalaceae family and the mechanisms underlying the anti-inflammatory properties of *P. tenuifolia*. This work is the first to report the inhibitory effects of extracts and isolated constituents of *P. tenuifolia* roots on the pro-inflammatory cytokines IL-12 p40, IL-6, and TNF-α. Aqueous fractionation of *P. tenuifolia* extracts is a promising area for further anti-inflammation research. Additional in vivo mechanistic studies will help determine the potential of phenolic glycosides and triterpenoid saponins for use as anti-inflammatory drugs to suppress inflammatory and related diseases.

## 3. Materials and Methods 

### 3.1. General Experimental Procedures

The optical rotation values were confirmed using a JASCO DIP-370 digital polarimeter (Hachioji, Tokyo, Japan). Electrospray ionization (ESI) mass spectra were obtained using an Agilent 1200 LC-MSD Trap spectrometer. LC-MS/MS analyses were performed using a Shimadzu LCMS-8040 system (Kyoto, Japan) in positive and negative mode. NMR spectra were analyzed on a JEOL ECA 400 and 600 spectrometer (JEOL Ltd, Tokyo, Japan) with TMS used as an internal standard. Sephadex LH-20 (GE Healthcare Bio-Science AB, Uppsala, Sweden) and Diaion HP-20 (Supelco, Bellefonte, PA, USA) resins were used. Thin layer chromatography (TLC) using YMC RP-18 resins was performed using pre-coated silica gel 60 F_254_ and RP-18 F_254S_ plates (both 0.25 mm, Merck, Darmstadt, Germany), and the spots were detected under UV light at 254 and 365 nm wavelengths and using 10% H_2_SO_4_, followed by heating for 3–5 min. The chemicals used were purchased from commercial suppliers and used as received. All chemical reagents were purchased from Sigma-Aldrich (St. Louis, MO, USA).

### 3.2. Plant Material

The root of *P. tenuifolia* was obtained from a herbal company. Plant identification was verified by an expert botanist (Y.H.K.). A representative specimen of *P. tenuifolia* (CNU PT 16005) was conserved in the Herbarium of the Natural Product Laboratory, Chungnam National University, Daejeon, Korea.

### 3.3. Extraction and Isolation

Dried roots of *P. tenuifolia* (2.5 kg) were extracted three times with methanol under reflux. The methanol extract (666.0 g) was suspended in H_2_O (3.0 L) and partitioned with dichloromethane and ethyl acetate to afford a dichloromethane fraction (D, 100.0 g), ethyl acetate fraction (E, 40.0 g), and water layer (W), respectively. The water layer (W) was passed through a Diaion HP-20 column and eluted with increasing concentrations of MeOH in water (0%, 25%, 50%, and 100%) to obtain three fractions (W1–W3) after removing the fraction that was eluted with water. Fraction W3 (250.0 g) was separated by medium-pressure liquid chromatography (MPLC) on a silica gel column using a mobile phase of CH_2_Cl_2_-MeOH-H_2_O (7:1:0.05, *v*/*v*) to obtain seven fractions (W3A–W3G). Fraction W3A (330.0 mg) was purified by YMC RP-18 CC using MeOH-H_2_O (2:1, *v*/*v*) as the eluent to furnish compounds **6** (7.5 mg) and **8** (10.3 mg). Fraction W3B (10.0 g) was rechromatographed by Sephadex LH-20 and RP-C18 CC with MeOH–H_2_O (1:1, *v*/*v*) to afford compounds **9** (6.8 mg), **1** (3.0 mg), **3** (5.0 mg), and **4** (8.9 mg). Fraction W3C (19.0 g) was separated on RP-C18 silica gel and Sephadex LH-20 to afford compounds **7** (25.0 mg), **5** (18.7 mg), and **2** (7.8 mg). Fraction W3G (28 g) was separated by YMC RP-18 and Sephadex LH-20 CC using solvent acetone−H_2_O (1:1, 2:1, and 3:1 *v*/*v*) and further purified by silica gel CC with CH_2_Cl_2_−MeOH−H_2_O (2.5:1:0.1, *v*/*v*) to afford compounds **10** (156.0 mg), **11** (220.3 mg) and **12** (188.9 mg). Repeating the same steps as for subfraction W3G, compounds **14** (266.6 mg) and **15** (15.3 mg) were obtained from subfraction W3F (16.0 g).

#### Physical Properties and Key Spectroscopic Data of Isolated Compounds:

Compound **1**. Yellow powder, ^1^H (400 MHz in MeOD-*d_4_*) *δ*_H_ 3.63 (d, *J* = 10.0 Hz), 3.67 (d, *J* = 9.0 Hz) and ^13^C-NMR (100 MHz in MeOD-*d_4_*): *δ*_C_ 65.6 (C-1), 104.9 (C-2), 79.8 (C-3), 74.1 (C-4), 84.2 (C-5), 63.4 (C-6), 93.2 (C-1’), 72.5 (C-2’), 74.9 (C-3’), 71.6 (C-4’), 72.5 (C-5’), 65.6 (C-6’), 131.6 (C-1’’), 106.9 (C-2’’), 154.9 (C-3’’), 141.4 (C-4’’), 154.9 (C-5’’), 106.9 (C-6’’), 147.4 (C-7’’), 117.8 (C-8’’), 168.2 (C-9’’), 56.8 (3’’, 5’’-OMe), 61.2 (4’’-OMe), 131.6 (C-1’’’), 131.3 (C-2’’’), 129.7 (C-3’’’), 134.4 (C-4’’’), 129.7 (C-5’’’), 130.8 (C-6’’’), 168.2 (C-7’’’).

Compound **2**. Yellow powder, ^1^H (400 MHz in MeOD-*d_4_*) *δ*_H_ 3.63 (d, *J* = 10.0 Hz), 3.67 (d, *J* = 9.0 Hz) and ^13^C-NMR (100 MHz in MeOD-*d_4_*): *δ*_C_ 63.3 (C-1), 104.8 (C-2), 79.7 (C-3), 74.9 (C-4), 84.1 (C-5), 63.3 (C-6), 93.3 (C-1’), 73.1 (C-2’), 74.9 (C-3’), 71.2 (C-4’), 73.8 (C-5’), 65.3 (C-6’), 127.8 (C-1’’), 112.1 (C-2’’), 149.5 (C-3’’), 150.8 (C-4’’), 116.5 (C-5’’), 124.2 (C-6’’), 115.1 (C-7’’), 147.8 (C-8’’), 168.5 (C-9’’), 56.9 (OMe).

Compound **3**. Yellow powder, ^1^H (400 MHz in MeOD-*d_4_*) *δ*_H_ 3.63 (d, *J* = 10.0 Hz), 3.67 (d, *J* = 9.0 Hz) and ^13^C-NMR (100 MHz in MeOD-*d_4_*): *δ*_C_ 65.3 (C-1), 104.8 (C-2), 79.7 (C-3), 74.6 (C-4), 84.1 (C-5), 63.3 (C-6), 93.3 (C-1’), 73.1 (C-2’), 74.9 (C-3’), 71.2 (C-4’), 73.8 (C-5’), 65.3 (C-6’), 127.8 (C-1’’), 112.1 (C-2’’), 149.5 (C-3’’), 150.8 (C-4’’), 116.5 (C-5’’), 124.3 (C-6’’), 115.1 (C-7’’), 147.8 (C-8’’), 168.5 (C-9’’), 56.5 (OMe).

Compound **4**. White amorphous powder, ^1^H (400 MHz in MeOD-*d_4_*) *δ*_H_ 3.63 (d, *J* = 10.0 Hz), 3.67 (d, *J* = 9.0 Hz) and ^13^C-NMR (100 MHz in MeOD-*d_4_*): *δ*_C_ 65.6, 104.9, 79.7, 74.0, 84.1, 63.4, 93.1, 73.1, 74.9, 71.6, 72.5, 65.3, 131.5, 106.9, 154.9, 141.4, 154.9, 106.9, 147.4, 117.8, 168.9, 56.7 (3’’, 5’’-OMe), 61.2 (4’’-OMe)

Compound **5**. White amorphous powder, ^1^H (400 MHz in MeOD-*d_4_*) *δ*_H_ 3.63 (d, *J* = 10.0 Hz), 3.67 (d, *J* = 9.0 Hz) and ^13^C-NMR (100 MHz in MeOD-*d_4_*): *δ*_C_ 65.6 (C-1), 104.8 (C-2), 79.3 (C-3), 74.1 (C-4), 84.2 (C-5), 63.7 (C-6), 92.6 (C-1’), 73.0 (C-2’), 75.0 (C-3’), 71.4 (C-4’), 72.4 (C-5’), 65.6 (C-6’), 126.5 (C-1’’), 106.9 (C-2’’), 154.3 (C-3’’), 139.5 (C-4’’), 149.3 (C-5’’), 106.9 (C-6’’), 147.4 (C-7’’), 115.7 (C-8’’), 168.3 (C-9’’), 56.7 (3’’-OMe), 126.5 (C-1’’’), 149.3 (C-2’’’), 149.3 (C-3’’’), 139.5 (C-4’’’), 149.3 (C-5’’’), 106.9 (C-6’’’), 147.4 (C-7’’’), 115.7 (C-8’’’), 169.2 (C-9’’’), 56.5 (OMe), 56.8 (OMe).

Compound **6**. Off-white solid, ^1^H (400 MHz in MeOD-*d_4_*) *δ*_H_ 3.86 (6H, s), 3.79(3H, s), and ^13^C-NMR (100 MHz in MeOD-*d_4_*): *δ*_C_ 65.3 (C-1), 104.8 (C-2), 79.8 (C-3), 74.6 (C-4), 84.2 (C-5), 63.7 (C-6), 92.6 (C-1’), 73.0 (C-2’), 75.0 (C-3’), 71.8 (C-4’), 72.4 (C-5’), 65.7 (C-6’), 131.3 (C-1’’), 106.9 (C-2’’), 154.6 (C-3’’), 141.2 (C-4’’), 154.6 (C-5’’), 106.8 (C-6’’), 147.3 (C-7’’), 117.6 (C-8’’), 167.9 (C-9’’), 56.7 (3’’, 5’’-OMe), 61.1 (4’’,-OMe), 123.4 (C-1’’’), 133.5(C-2’’’), 114.9 (C-3’’’), 165.3 (C-4’’’), 114.9 (C-5’’’), 132.9 (C-6’’’), 167.8 (C-7’’’), 56.0 (OMe).

Compound **7**. White amorphous powder, ^1^H (400 MHz in MeOD-*d_4_*) *δ*_H_ 3.63 (d, *J* = 10.0 Hz), 3.67 (d, *J* = 9.0 Hz) and ^13^C-NMR (100 MHz in MeOD-*d_4_*): *δ*_C_ 65.6 (C-1), 104.9 (C-2), 79.3 (C-3), 74.2 (C-4), 84.3 (C-5), 63.9 (C-6), 92.7 (C-1’), 73.1 (C-2’), 75.0 (C-3’), 71.9 (C-4’), 72.4 (C-5’), 65.6 (C-6’), 127.7 (C-1’’), 112.1 (C-2’’), 149.5 (C-3’’), 150.8 (C-4’’), 116.5 (C-5’’), 124.3 (C-6’’), 147.4 (C-7’’), 115.0 (C-8’’), 168.5 (C-9’’), 56.5 (3’’-OMe), 126.6 (C-1’’’), 106.9 (C-2’’’), 149.5 (C-3’’’), 139.6 (C-4’’’), 149.5 (C-5’’’), 106.9 (C-6’’’), 115.8 (C-7’’’), 147.4 (C-8’’’), 169.2 (C-9’’’), 56.8 (OMe).

Compound **8**. Yellow powder, ^1^H (400 MHz in MeOD-*d_4_*) *δ*_H_ 3.63 (d, *J* = 10.0 Hz), 3.67 (d, *J* = 9.0 Hz) and ^13^C-NMR (100 MHz in MeOD-*d_4_*): *δ*_C_ 65.5 (C-1), 104.8 (C-2), 79.3 (C-3), 74.1 (C-4), 84.2 (C-5), 63.7 (C-6), 92.6 (C-1’), 72.9 (C-2’), 75.0 (C-3’), 71.8 (C-4’), 72.4 (C-5’), 65.6 (C-6’), 131.3 (C-1’’), 106.8 (C-2’’), 154.6 (C-3’’), 141.2 (C-4’’), 154.6 (C-5’’), 106.8 (C-6’’), 147.4 (C-7’’), 117.6 (C-8’’), 167.9 (C-9’’), 56.7 (3’’, 5’’-OMe), 61.2 (4’’-OMe), 126.5 (C-1’’’), 106.8 (C-2’’’), 149.3 (C-3’’’), 139.4 (C-4’’’), 149.3 (C-5’’’), 106.8 (C-6’’’), 169.2 (C-7’’’), 115.7 (C-8’’’), 147.3 (C-9’’’), 56.5 (OMe), 56.8 (OMe).

Compound **9**. Yellow powder, ^1^H (400 MHz in MeOD-*d_4_*) *δ*_H_ 3.63 (d, *J* = 10.0 Hz), 3.67 (d, *J* = 9.0 Hz) and ^13^C-NMR (100 MHz in MeOD-*d_4_*): *δ*_C_ 65.5 (C-1), 104.8 (C-2), 79.3 (C-3), 74.1 (C-4), 84.2 (C-5), 63.7 (C-6), 92.6 (C-1’), 72.9 (C-2’), 75.0 (C-3’), 71.8 (C-4’), 72.4 (C-5’), 65.6 (C-6’), 131.3 (C-1’’), 106.8 (C-2’’), 154.6 (C-3’’), 141.2 (C-4’’), 154.6 (C-5’’), 106.8 (C-6’’), 147.4 (C-7’’), 117.6 (C-8’’), 167.9 (C-9’’), 56.7 (3’’, 5’’-OMe), 61.2 (4’’-OMe), 126.5 (C-1’’’), 106.8 (C-2’’’), 149.3 (C-3’’’), 139.4 (C-4’’’), 149.3 (C-5’’’), 106.8 (C-6’’’), 169.2 (C-7’’’), 115.7 (C-8’’’), 147.3 (C-9’’’), 56.5 (OMe), 56.8 (OMe).

Compound **10**. White amorphous powder, ^1^H (600 MHz in MeOD-*d_4_*) *δ*_H_ 5.04 (d, *J* = 8.0 Hz), 6.08 (d, *J* = 8.0 Hz), 5.81 (br, s), 5.56 (br, s), 5.26 (d, *J* = 7.5 Hz), 6.09 (d, *J* = 3.0 Hz), 5.16 (d, *J* = 7.0 Hz), and ^13^C-NMR (150 MHz in MeOD-*d_4_*): *δ*_C_ 105.4 (C-1’, Glc), 95.0 (C-1’’, Fuc), 102.2 (C-1’’’, Rha-1), 104.9 (C-1’’’’, Rha-2), 104.8 (C-1’’’’’’, Xyl), 111.9 (C-1’’’’’’’’, Api), 105.5 (C-1’’’’’’’’’, Ara); LC-MS *m/z* 1697.8 [M + Na]^+^ (calcd for C_79_H_118_NaO_38_^+^, 1697.7), and *m/z* 1673.9 [M − H]^−^ (calcd for C_79_H_117_O_38_^−^, 1673.7).

Compound **11**. White amorphous powder, ^1^H (600 MHz in MeOD-*d_4_*) *δ*_H_ 4.93 (d, *J* = 7.8 Hz), 5.78 (d, *J* = 8.0 Hz), 6.23 (br, s), 4.77 (d, *J* = 7.5 Hz), 4.83 (d, *J* = 7.0 Hz), and ^13^C-NMR (150 MHz in MeOD-*d_4_*): *δ*_C_ 103.9 (C-1’, Glc), 94.2 (C-1’’, Fuc), 100.9 (C-1’’’, Rha-1), 106.3 (C-1’’’’, Xyl), 103.3 (C-1’’’’’, Gal); LC-MS *m/z* 1289.5 [M + Na]^+^ (calcd for C_59_H_94_NaO_29_^+^, 1289.5), and *m/z* 1265.8 [M − H]^−^ (calcd for C_59_H_93_O_29_^−^, 1265.5).

Compound **12**. White amorphous powder, ^1^H (600 MHz in MeOD-d_4_) *δ*_H_ 6.64 (d, *J* = 16.0 Hz), 7.13 (d, *J* = 9.0 Hz), 7.74 (d, *J* = 8.9 Hz), 7.88 (d, *J* = 16.0 Hz), and ^13^C-NMR (150 MHz in MeOD-d_4_): *δ*_C_ 105.0 (C-1’, Glc), 94.9 (C-1’’, Fuc), 101.8 (C-1’’’, Rha-1), 106.6 (C-1’’’’, Xyl), 104.2 (C-1’’’’’, Gal) 104.5 (C-1’’’’’’, Rha-2); LC-MS *m/z* 1595.7 [M + Na]^+^ (calcd for C_75_H_112_NaO_35_^+^, 1595.6), and *m/z* 1571.8 [M − H]^−^ (calcd for C_75_H_111_O_35_^−^, 1571.6).

Compound **13**. White amorphous powder, ^1^H (600 MHz in MeOD-*d_4_*) *δ*_H_ 5.01 (d, *J* = 8.0 Hz), 5.86 (d, *J* = 8.0 Hz), 6.32 (br, s), 4.81 (d, *J* = 7.5 Hz), 4.87 (d, *J* = 8.0 Hz), and ^13^C-NMR (150 MHz in MeOD-*d_4_*): *δ*_C_ 104.1 (C-1’, Glc), 94.0 (C-1’’, Fuc), 100.9 (C-1’’’, Rha), 106.3 (C-1’’’’, Xyl), 106.3 (C-1’’’’’, Gal); LC-MS *m/z* 1268.7 [M+NH_4_]^+^ (calcd for C_59_H_98_NO_28_^+^, 1268.6), and *m/z* 1249.9 [M − H]^−^ (calcd for C_57_H_91_O_28_^−^, 1249.5).

Compound **14**. White amorphous powder, ^1^H (600 MHz in MeOD-*d_4_*) *δ*_H_ 5.06 (d, *J* = 8.0 Hz), 6.06 (d, *J* = 8.0 Hz), δ_H_ 6.40 (br s), 5.40 (d, *J* = 7.0 Hz), and ^13^C-NMR (150 MHz in MeOD-d4): *δ*_C_ 105.4 (C-1’, Glc), 94.8 (C-1’’, Fuc), 101.2 (C-1’’’, Rha), 107.4 (C-1’’’’, Xyl); LC-MS *m/z* 1127.5 [M + Na]^+^ (calcd for C_53_H_84_NaO_24_^+^, 1127.5), and *m/z* 1103.7 [M − H]^−^ (calcd for C_53_H_83_O_24_^−^, 1103.7).

Compound **15**. White amorphous powder, ^1^H (600 MHz in C_5_D_5_N-*d_5_*) *δ*_H_ 5.09 (d, *J* = 7.5 Hz), 5.67 (d, *J* = 3.7 Hz), δ_H_ 5.84 (d, *J* = 1.6 Hz), 6.26 (d, *J* = 2.4 Hz), δ_H_ 6.45 (d, *J* = 3.3 Hz), and ^13^C-NMR (150 MHz in C_5_D_5_N-*d_5_*): *δ*_C_ 105.9 (C-1’, Glc), 93.7 (C-1’’, Ara), 101.0 (C-1’’’, Rha), 106.6 (C-1’’’’, Xyl), Api (C-1’’’’’); LC-MS *m/z* 1247.6 [M + Na]^+^ (calcd for C_57_H_92_NaO_28_^+^, 1247.5), and *m/z* 1223.7 [M − H]^−^ (calcd for C_57_H_91_O_28_^−^, 1223.5).

### 3.4. Cell Culture and Reagents

BMDCs were grown from wild-type C57BL/6 mice (Orient Bio Inc., Seoul, Korea) as previously described [18,23]. All animal procedures were approved by and performed according to the guidelines of the Institutional Animal Care and Use Committee of Jeju National University (#2016-0059). Briefly, bone marrow from the tibia and femur was obtained by flushing with Dulbecco’s Modified Eagle Medium (DMEM; Welgene, Gyeongsan, Korea) and bone marrow cells were cultured in RPMI 1640 medium containing 10% heat-inactivated fetal bovine serum (FBS; Gibco, New York, NY, USA), 50 μM of 2-ME, and 2 mM of glutamine, supplemented with 3% J558L hybridoma cell culture supernatant containing granulocyte-macrophage colony-stimulating factor (GM-CSF). The culture medium containing GM-CSF was replaced every other day. At day 6 of culture, non-adherent cells and loosely adherent DC aggregates were harvested, washed, and resuspended in RPMI 1640, supplemented with 5% FBS. DCs were incubated in 48-well plates at a density of 1 × 10^5^ cells/0.5 mL and then treated with the isolated compounds at the indicated concentration for 1 h before stimulation with 10 ng/mL of LPS from *Salmonella minnesota* (Alexis, New York, USA). Supernatants were harvested 18 h after stimulation. Concentrations of murine IL-12 p40, IL-6, and TNF-α in the culture supernatants were determined by ELISA (BD PharMingen, San Diego, CA, USA) according to the manufacturer’s protocols. All experiments were performed at least three times. Data are presented as the mean and the standard deviation (SD) of three independent experiments.

### 3.5. Cytokine Production Measurements

The BMDCs were incubated in 48-well plates in 0.5 mL containing 1 × 10^5^ cells per well, and then treated with isolated compounds **1**−**15** at the indicated concentration for 1 h before stimulation with 10 ng/mL LPS from *Salmonella minnesota* (Alexis, NY, USA) Supernatants were collected 18 h after stimulation. Concentrations of murine IL-12 p40, IL-6, and TNF-α in the culture supernatants were identified by ELISA (BD PharMingen, CA, USA) according to the manufacturer’s instructions.

The inhibitory activity (I) was expressed as the inhibition rate (%), which was calculated using the following formula:

$$I=\frac{\mathrm{Cdvc}-\mathrm{Cdcc}}{\mathrm{Cdvc}}\times 100$$

where Cdvc is the cytokine level (ng/mL) in vehicle-treated DC, and Cdcc is the cytokine level (ng/mL) in compound-treated DC. The data were obtained by at least three independent experiments performed in triplicate.

### 3.6. Cell Viability Assay

To evaluate the effects of isolated compounds on cell viability, we conducted an MTT assay [18,24]. BMDCs were incubated with 1 to 50 µM of isolated compounds for 18 h. The results demonstrate that compounds **1**, **2**, and **13** showed strong cytotoxicity toward BMDCs. Other compounds displayed no notable cytotoxicity against BMDCs.

### 3.7. Statistical Analysis

All results are presented as means ± SD. Data were analyzed by one-factor analysis of variance (ANOVA). * *P* value < 0.05, and ** *P* value < 0.01 were considered statistically significant. All experiments were repeated at least three times independently.

## Figures and Tables

**Table 1:** Anti-inflammatory effects of extracts of *P. tenuifolia* roots on LPS-stimulated bone marrow-derived dendritic cells.

Extracts		IC_50_ ± SD (µg/mL) ^a^		
		IL-12 p40	IL-6	TNF-α
1	**MeOH extract**	3.38 ± 0.02	1.65 ± 0.16	3.09 ± 0.14
2	**Water-layer**	0.94 ± 0.04	0.24 ± 0.08	2.43 ± 0.22
3	**EtOAc fraction**	0.05 ± 0.01	5.83 ± 0.26	3.92 ± 0.13
4	**CH_2_Cl_2_ fraction**	0.37 ± 0.10	0.89 ± 0.12	0.005 ± 0.001
**SB203580 ^b^**		5.00 ± 0.08	3.50 ± 0.08	7.20 ± 0.06

^a^ IC_50_ values < 50 µg/mL are considered to be active. ^b^ Positive control

**Table 2:** Anti-inflammatory effects of isolated compounds from *P. tenuifolia* roots on LPS-stimulated bone marrow-derived dendritic cells.

Compounds		IC_50_ (µM) ^a^		
		IL-12 p40	IL-6	TNF-α
**3**		9.25 ± 0.06	7.54 ± 0.08	5.77 ± 0.12
**4**		6.21 ± 0.27	6.44 ± 0.32	1.50 ± 0.25
**5**		14.34 ± 0.03	2.36 ± 0.08	1.04 ± 0.12
**6**		0.55 ± 0.03	0.35 ± 0.03	1.97 ± 0.03
**7**		2.99 ± 0.60	3.19 ± 0.03	6.08 ± 0.10
**8**		6.65 ± 0.03	5.82 ± 0.03	3.51 ± 0.03
**9**		5.89 ± 0.08	4.64 ± 0.08	5.08 ± 0.10
**10**		9.78 ± 0.09	9.04 ± 0.05	2.99 ± 0.29
**11**		21.05 ± 0.40	2.42 ± 0.03	6.34 ± 0.12
**12**		0.43 ± 0.05	0.83 ± 0.09	1.75 ± 0.02
**14**		10.52 ± 0.10	6.05 ± 0.03	2.05 ± 0.02
**15**		0.08 ± 0.01	0.24 ± 0.06	1.17 ± 0.13
**SB203580 ^b^**		5.00 ± 0.01	3.50 ± 0.02	7.20 ± 0.02

^a^ IC_50_ values < 50 µM are considered to be active. ^b^ Positive control.

## Supplementary Materials

The following are available online at https://www.mdpi.com/2223-7747/9/9/1240/s1, Figures S1–S54: 1D (^1^H, ^13^C-NMR) and 2D (COSY, HMQC, and HMBC) spectra, as well as LC-MS data of all isolated compounds.
