# Supplementary material for: Bioactive Compounds from Polygala tenuifolia and Their Inhibitory Effects on Lipopolysaccharide-Stimulated Pro-inflammatory Cytokine Production in Bone Marrow-Derived Dendritic Cells
Source: Plants (Basel). 2020 Sep 20;9(9):1240. doi: 10.3390/plants9091240 (PMC7570142; doi:10.3390/plants9091240)
Supplement: Supplementary file 1 [file plants-09-01240-s001.pdf]

SUPPLEMENTARY MATERIAL

Bioactive Compounds from *Polygala tenuifolia* and Their Inhibitory Effects on Lipopolysaccharide-Stimulated Pro-inflammatory Cytokine Production in Bone Marrow-Derived Dendritic Cells

Le Ba Vinh [1,2,†], Myungsook Heo [1,†], Nguyen Viet Phong [2], Irshad Ali [3], Young Sang Koh [3], Young Ho Kim [1,*], Seo Young Yang [1,*]

[1] College of Pharmacy, Chungnam National University, Daejeon 34134, Republic of Korea; vinhrooney@gmail.com (L.B.V.); inyl1110@naver.com (M.H.)

[2] Institute of Marine Biochemistry (IMBC), Vietnam Academy of Science and Technology (VAST), Hanoi 100000, Vietnam; vinhrooney@gmail.com (L.B.V.); ngvietphong@gmail.com (N.V.P.)

[3] School of Medicine and Jeju Research Center for Natural Medicine, Jeju National University, Jeju 63243, Korea; irshad.qau200@gmail.com (I.A.); yskoh7@jejunu.ac.kr (Y.S.K.)

† These authors contributed equally to this work.

* Correspondence: yhk@cnu.ac.kr (Y.H.K.); syyang@cnu.ac.kr (S.Y.Y.); Tel.: +82-42-821-5933 (Y.H.K.); +82-42-821-7321 (S.Y.Y.)

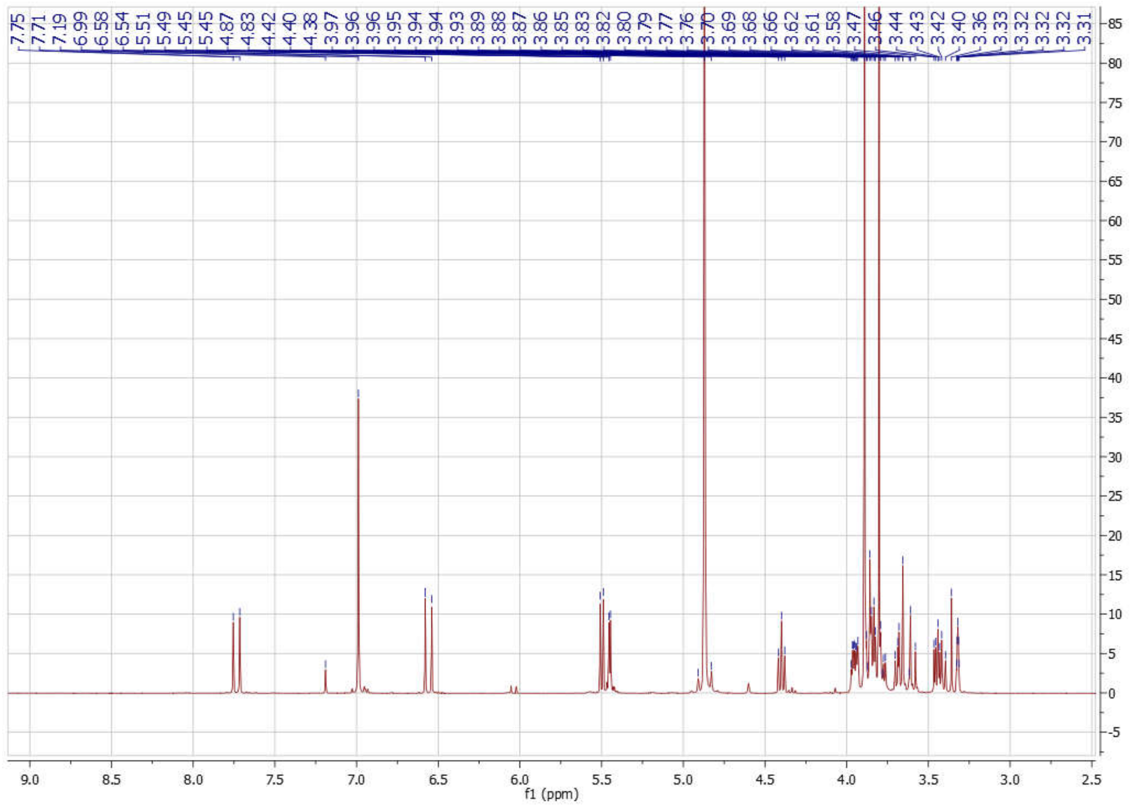

Figure S1. ^{1}H-NMR spectrum (MeOD, 400 MHz) of compound (**1**)

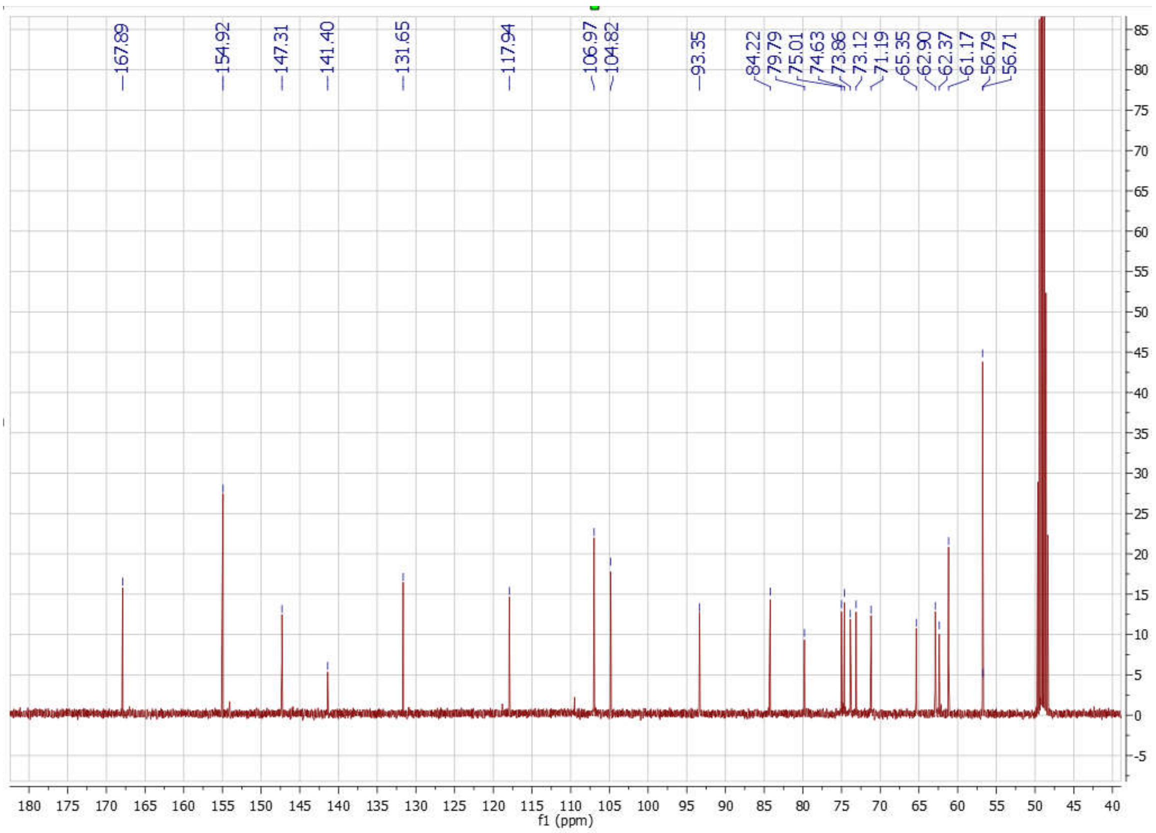

Figure S2. ^{13}C-NMR spectrum (MeOD, 100 MHz) of compound (**1**)

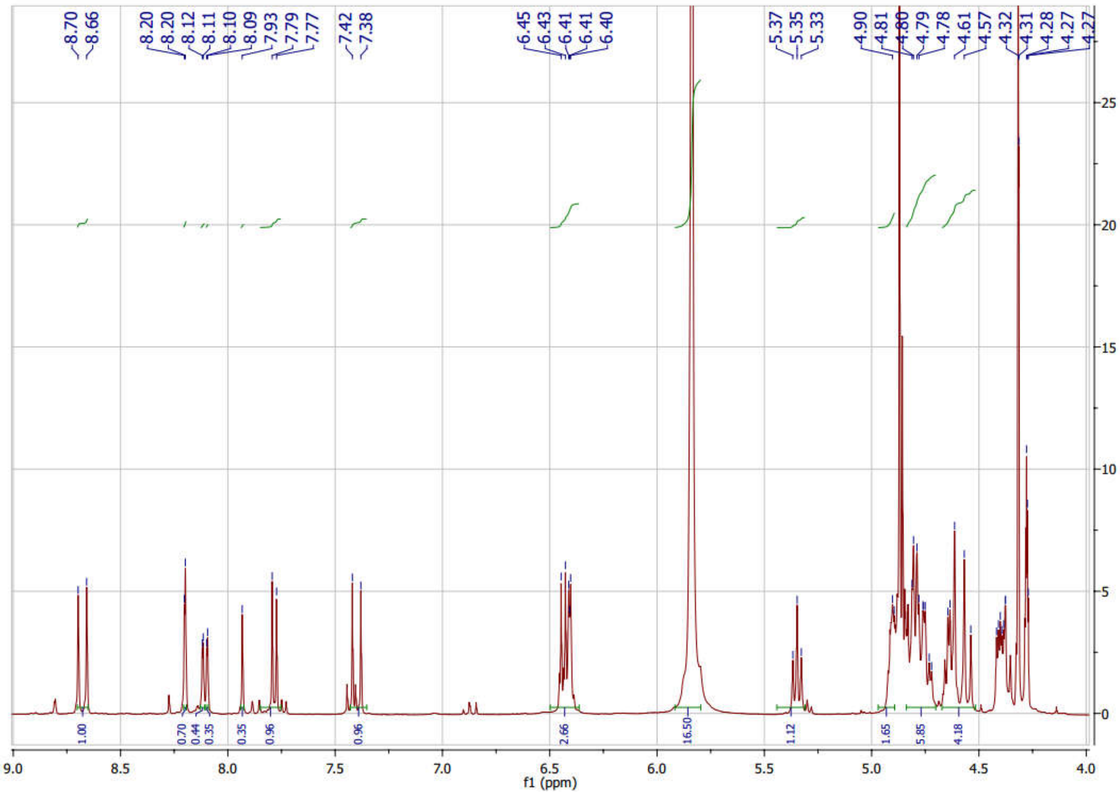

Figure S3. ^{1}H-NMR spectrum (MeOD, 400 MHz) of compound (**2**)

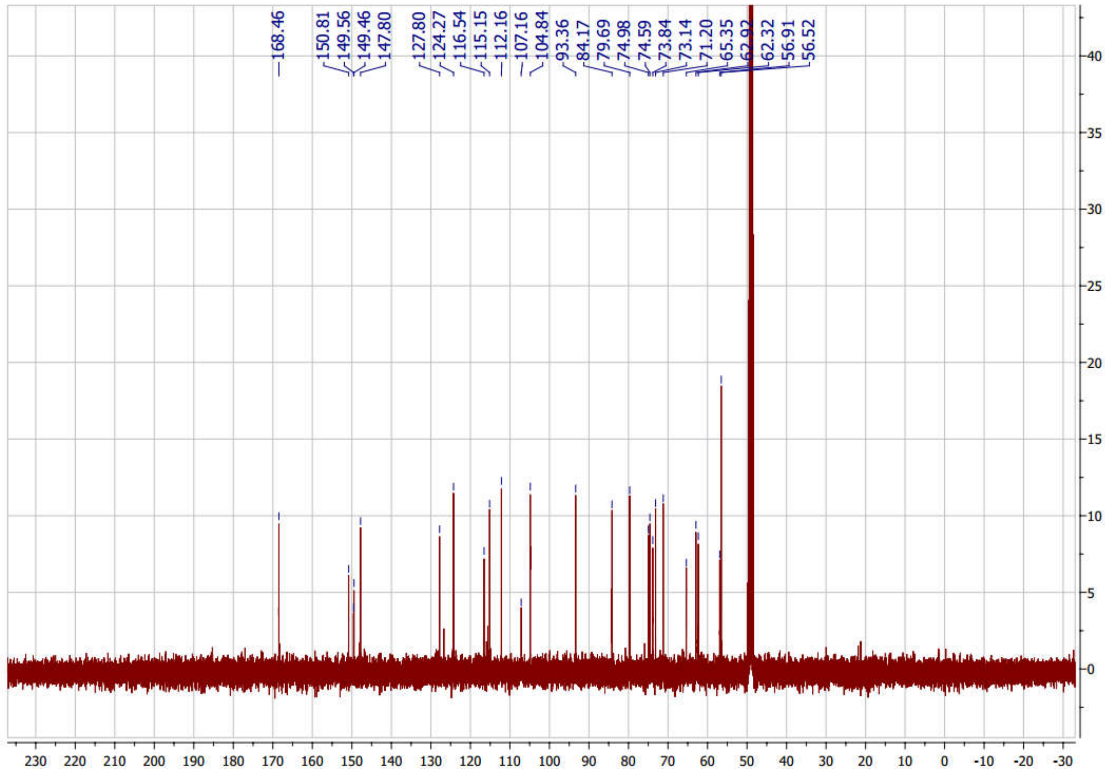

Figure S4. ^{13}C-NMR spectrum (MeOD, 100 MHz) of compound (**2**)

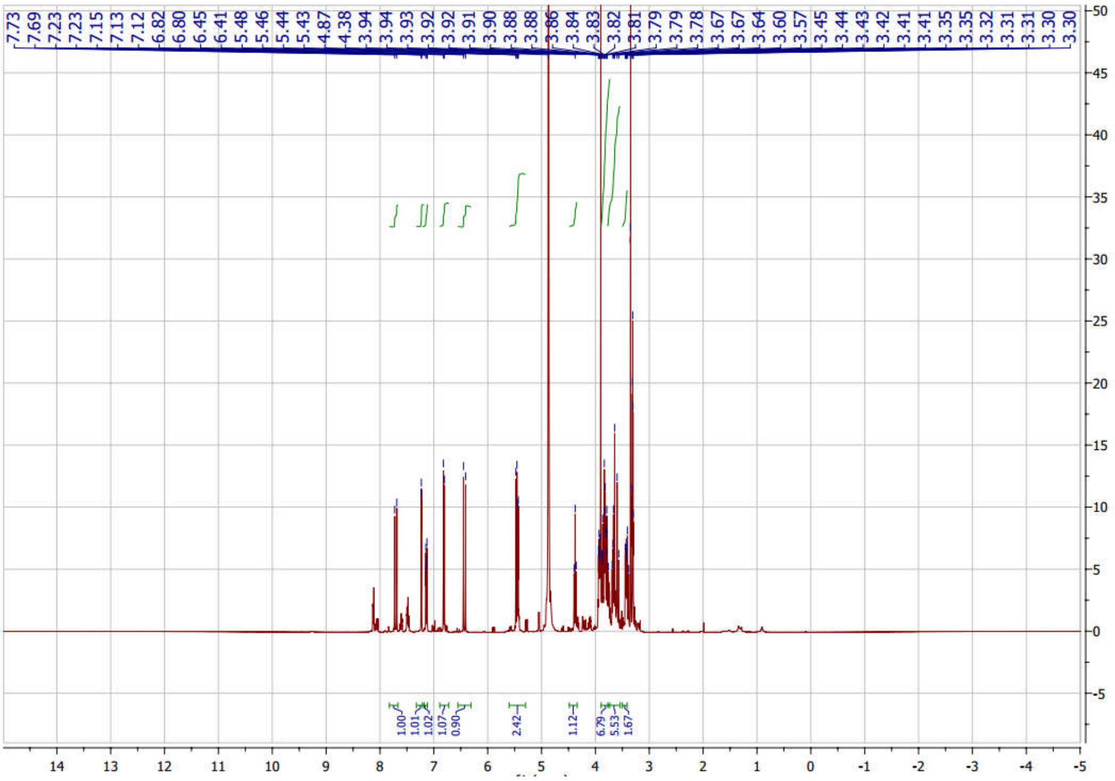

Figure S5. ^{1}H-NMR spectrum (MeOD, 400 MHz) of compound (**3**)

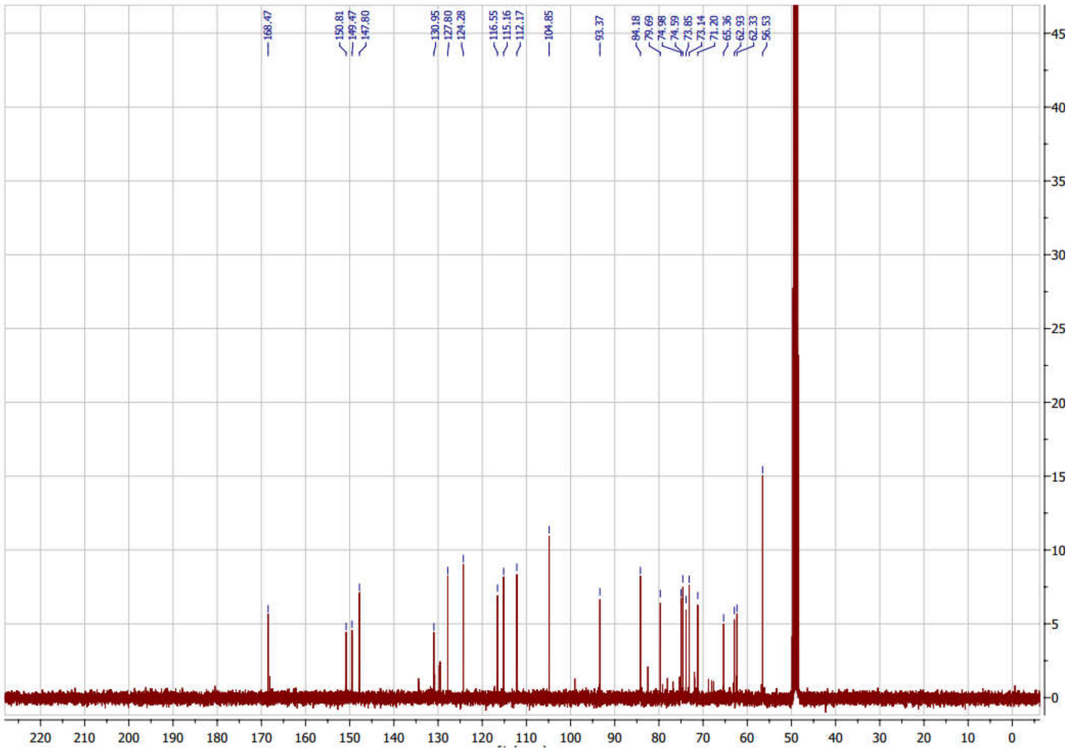

Figure S6. ^{13}C-NMR spectrum (MeOD, 100 MHz) of compound (**3**)

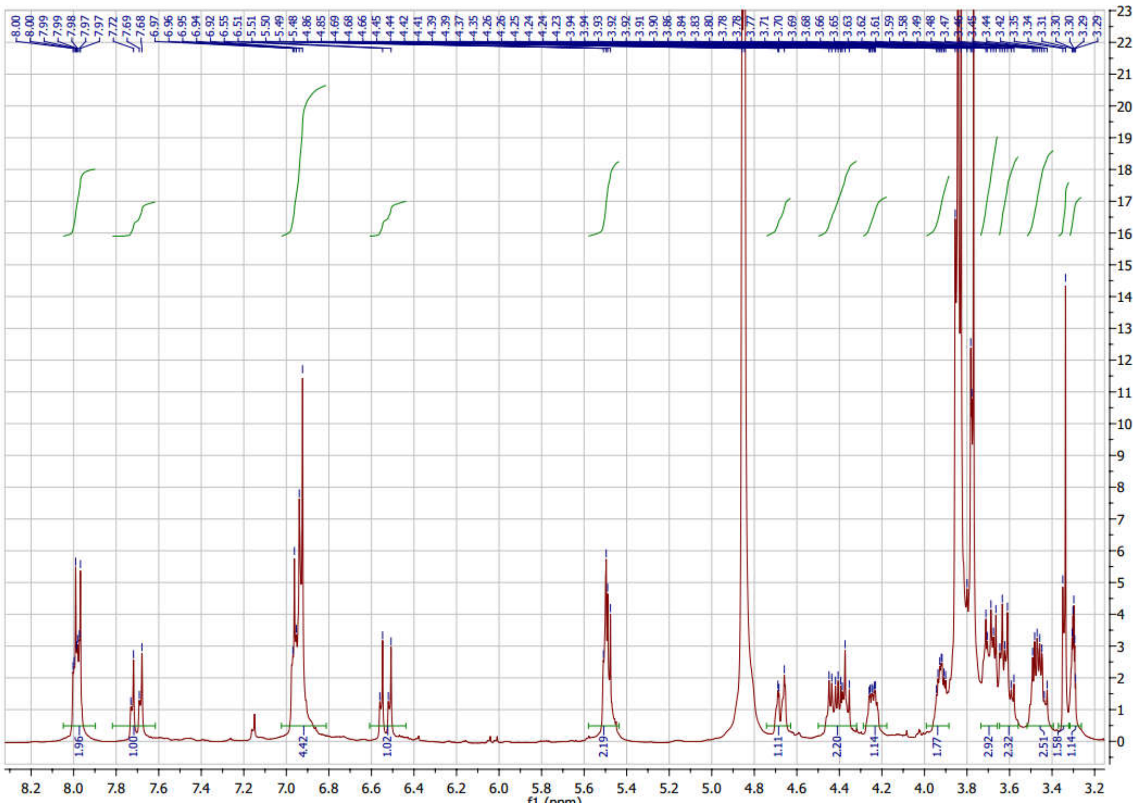

Figure S7. ^{1}H-NMR spectrum (MeOD, 400 MHz) of compound (**4**)

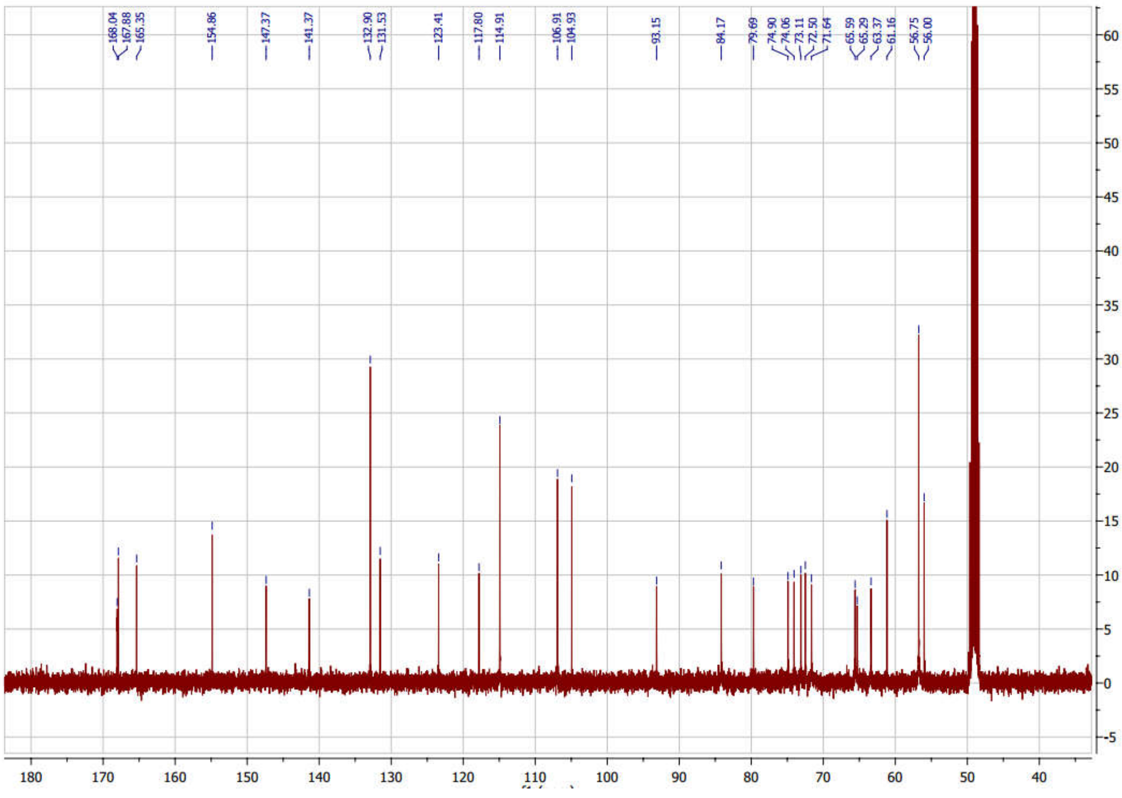

Figure S8. ^{13}C-NMR spectrum (MeOD, 400 MHz) of compound (**4**)

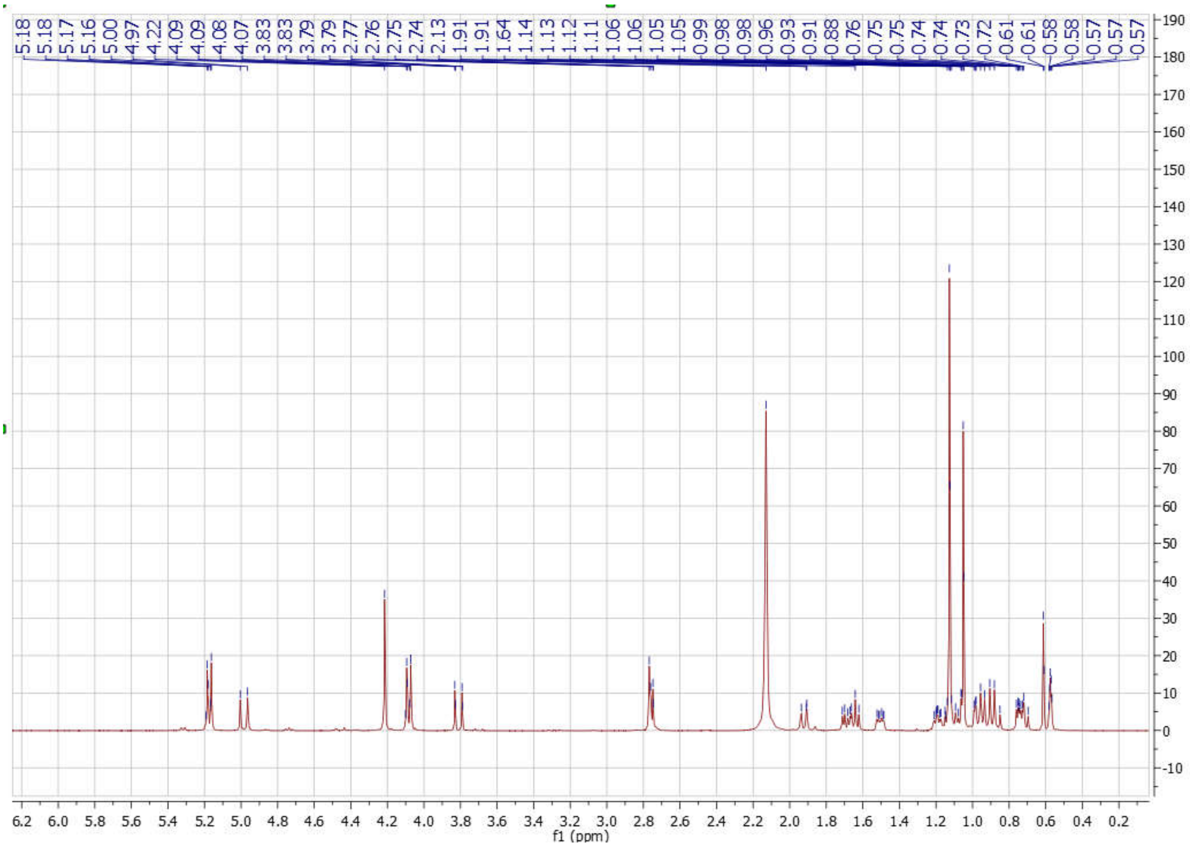

Figure S9. ^{1}H-NMR spectrum (MeOD, 400 MHz) of compound (**5**)

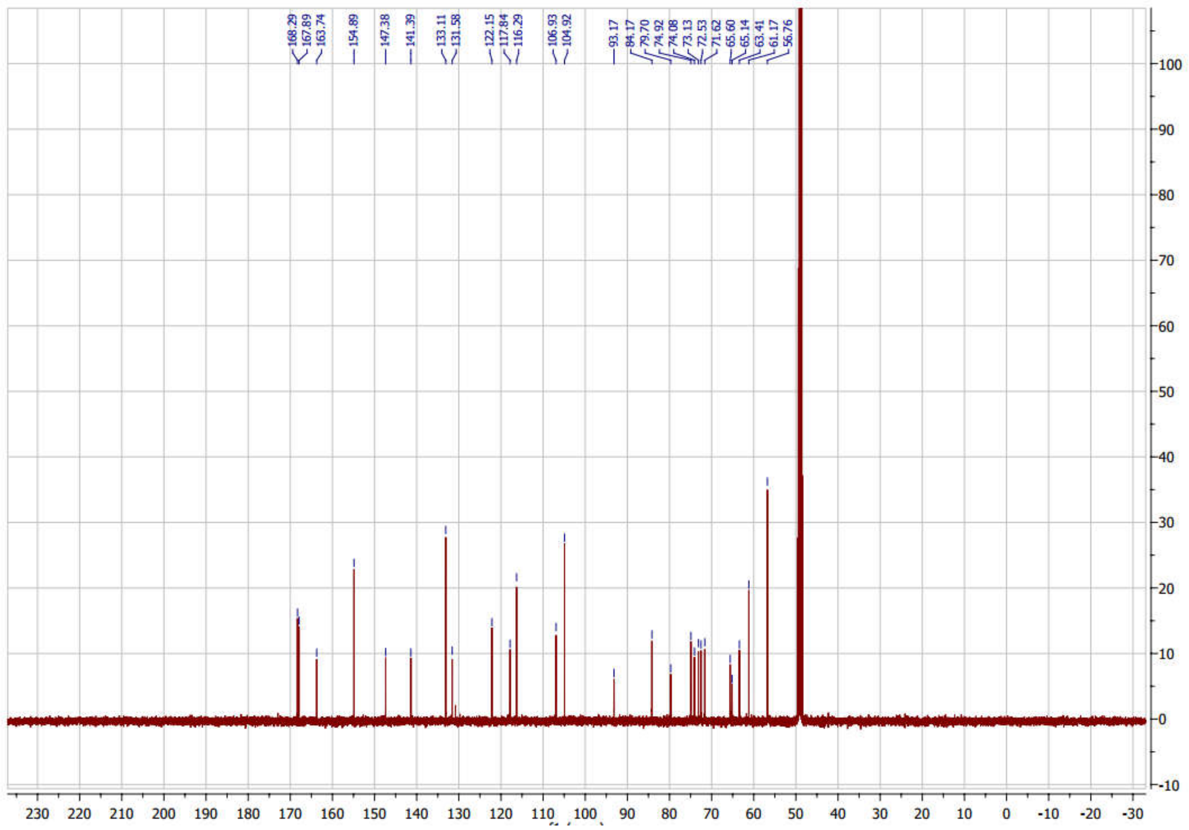

Figure S10. ^{13}C-NMR spectrum (MeOD, 100 MHz) of compound (**5**)

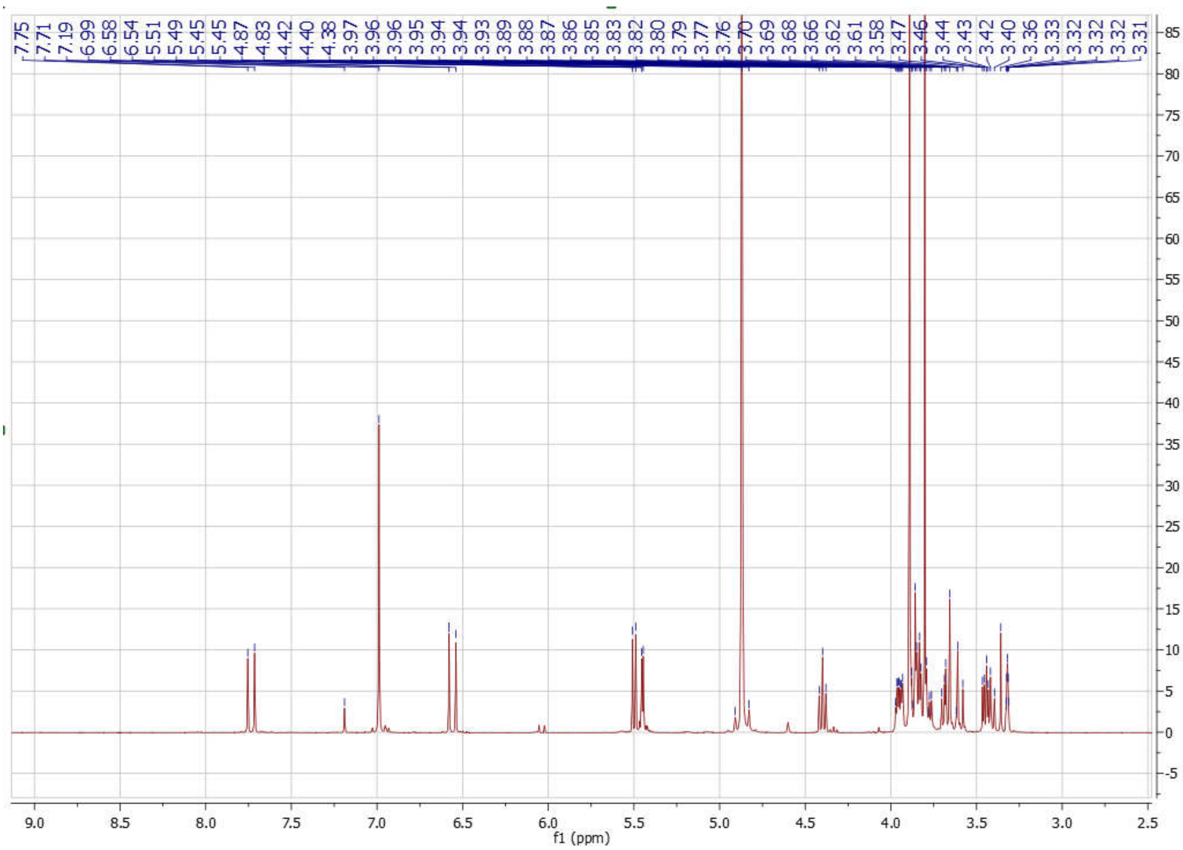

Figure S11. ^{1}H-NMR spectrum (MeOD, 400 MHz) of compound (**6**)

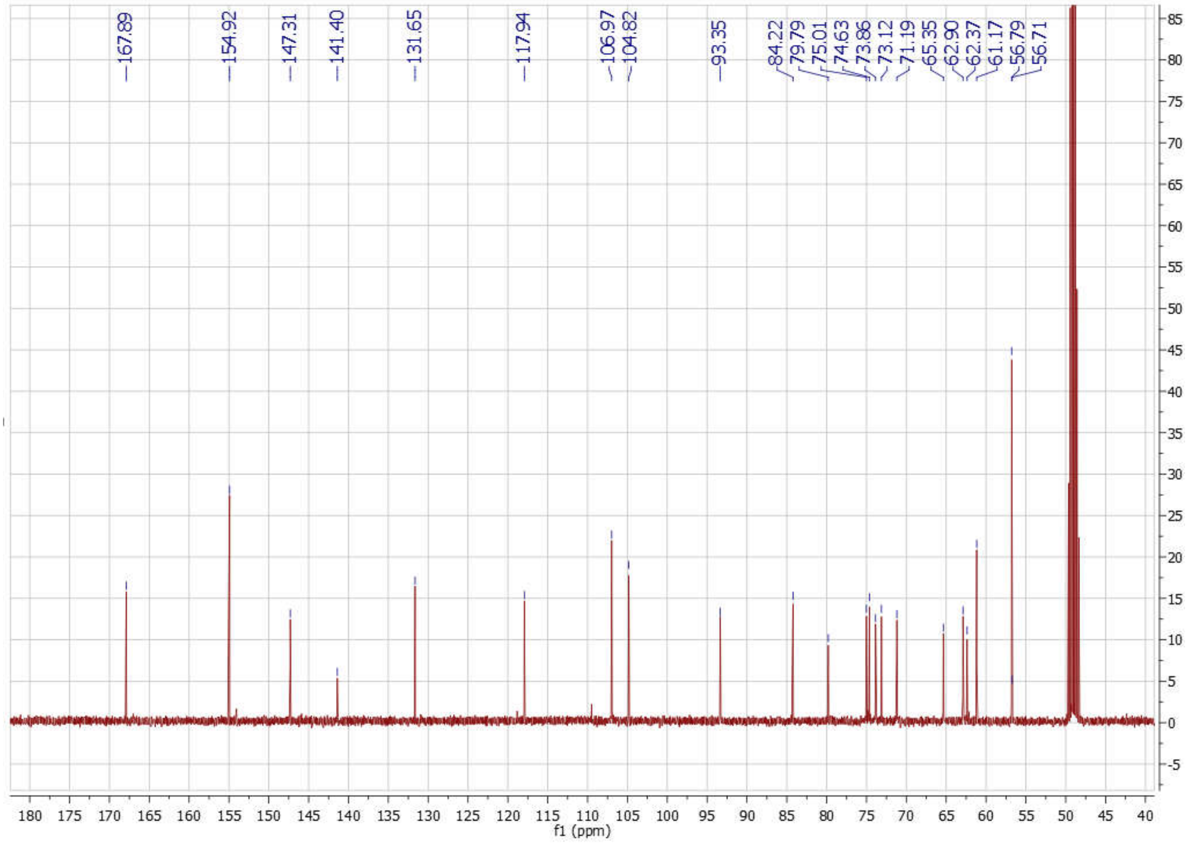

Figure S12. ^{13}C-NMR spectrum (MeOD, 100 MHz) of compound (**6**)

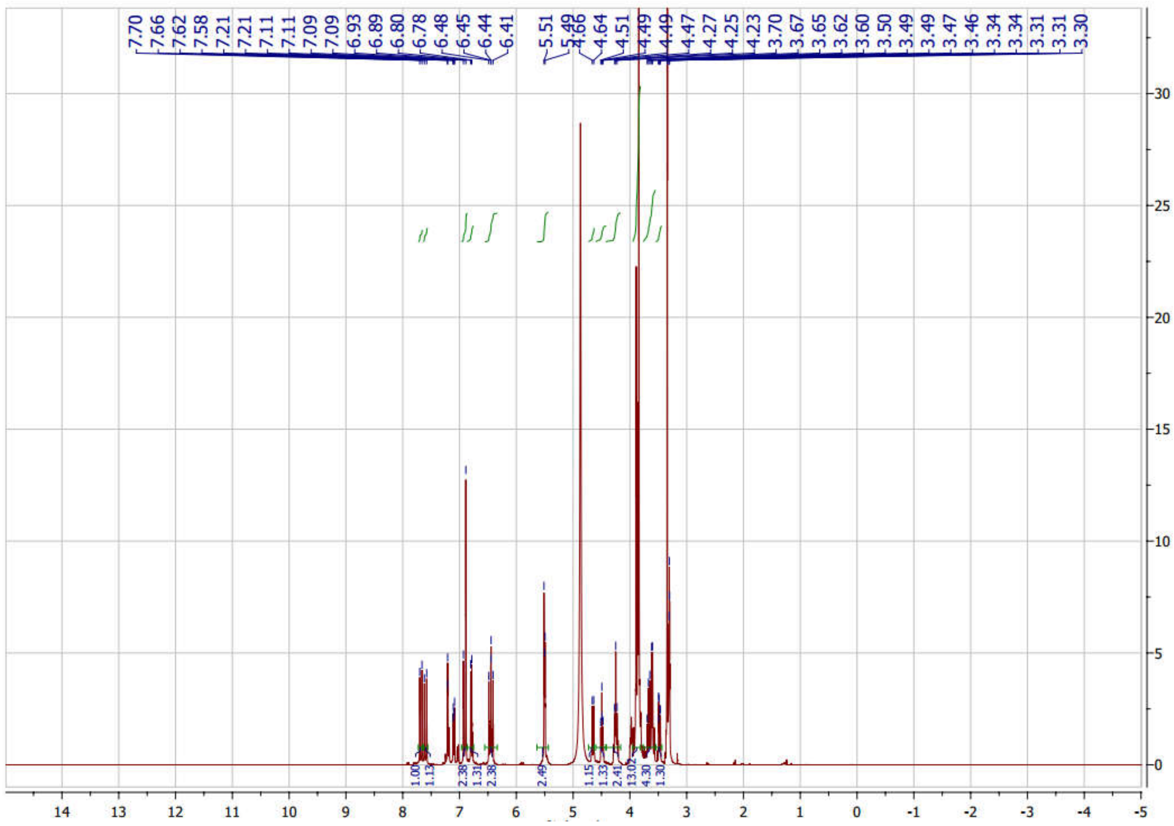

Figure S13. ^{1}H-NMR spectrum (MeOD, 400 MHz) of compound (**7**)

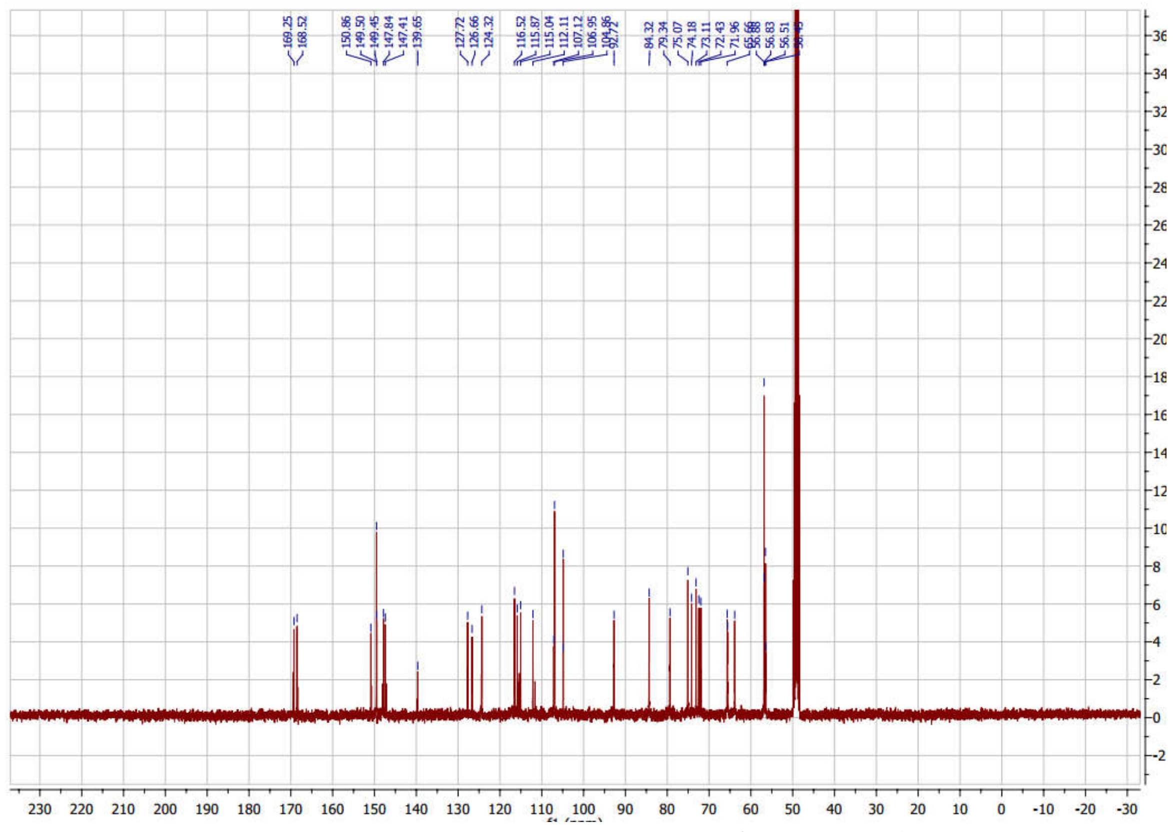

Figure S14. ^{13}C-NMR spectrum (MeOD, 100 MHz) of compound (**7**)

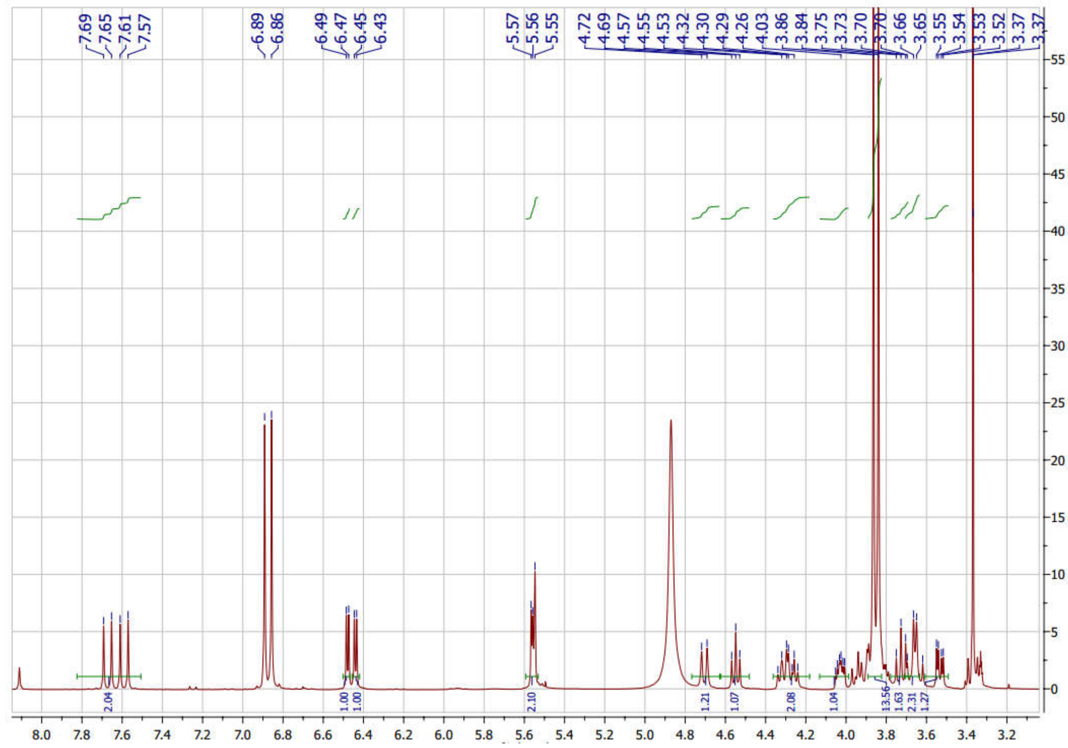

Figure S15. ^{1}H-NMR spectrum (MeOD, 400 MHz) of compound (**8**)

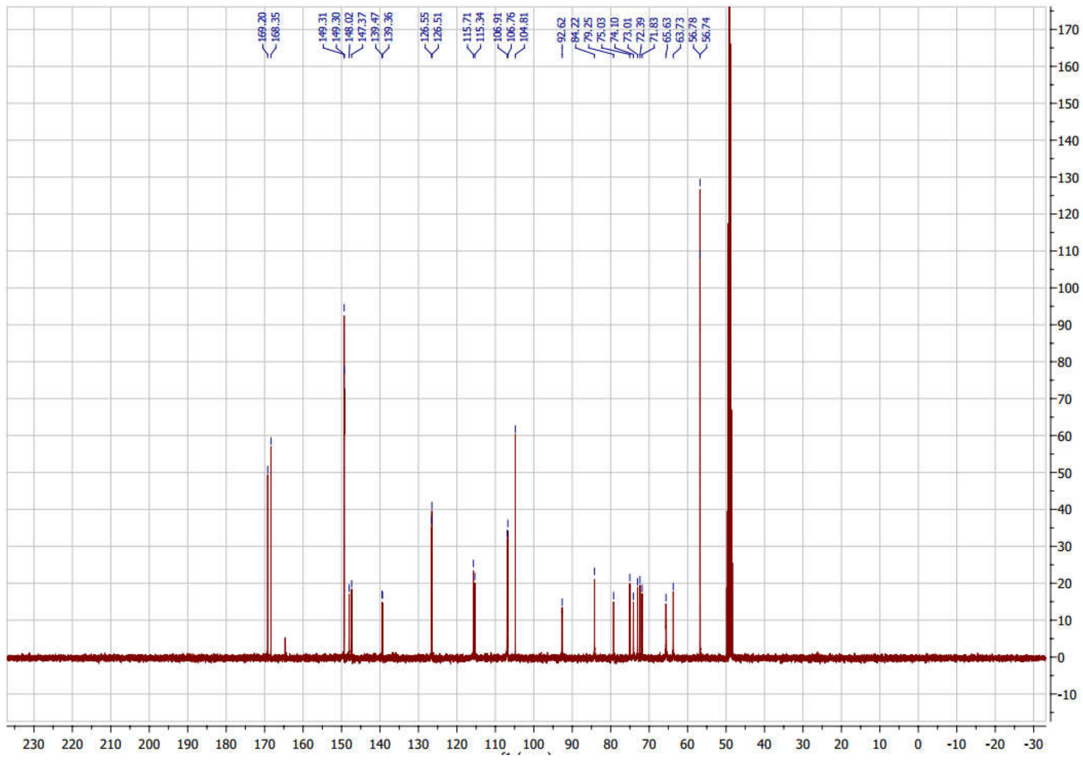

Figure S16. ^{13}C-NMR spectrum (MeOD, 100 MHz) of compound (**8**)

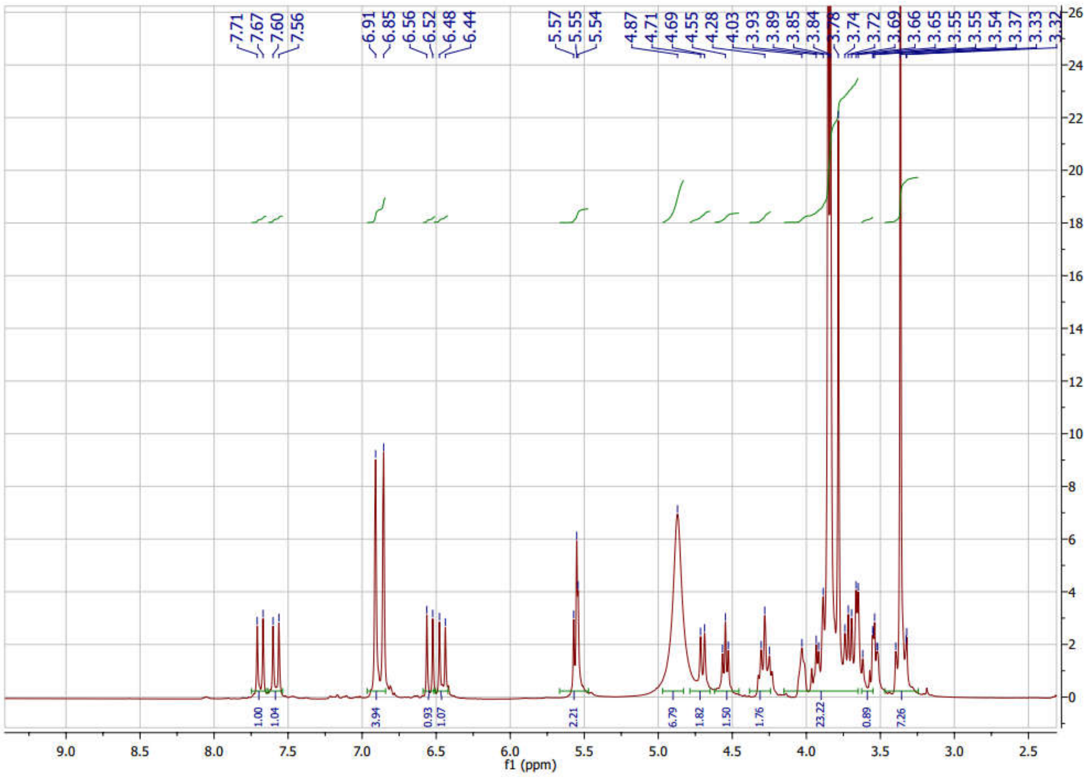

Figure S17. ^{1}H-NMR spectrum (MeOD, 400 MHz) of compound (**9**)

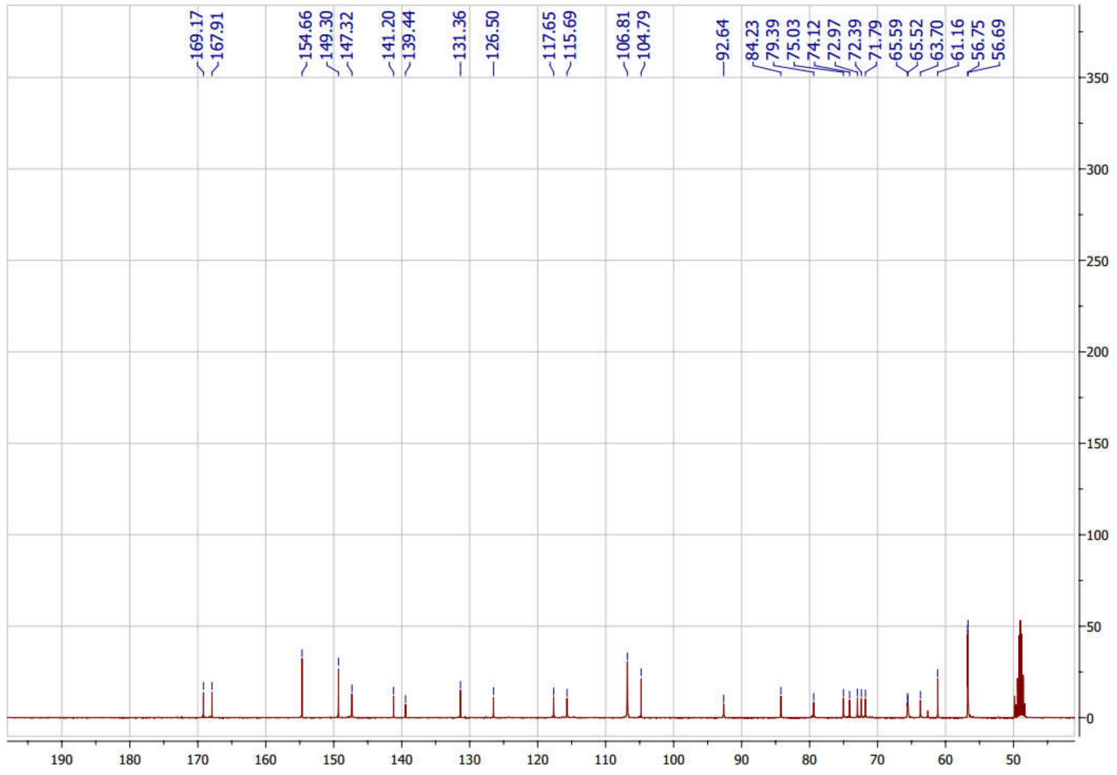

Figure S18. ^{13}C-NMR spectrum (MeOD, 100 MHz) of compound (**9**)

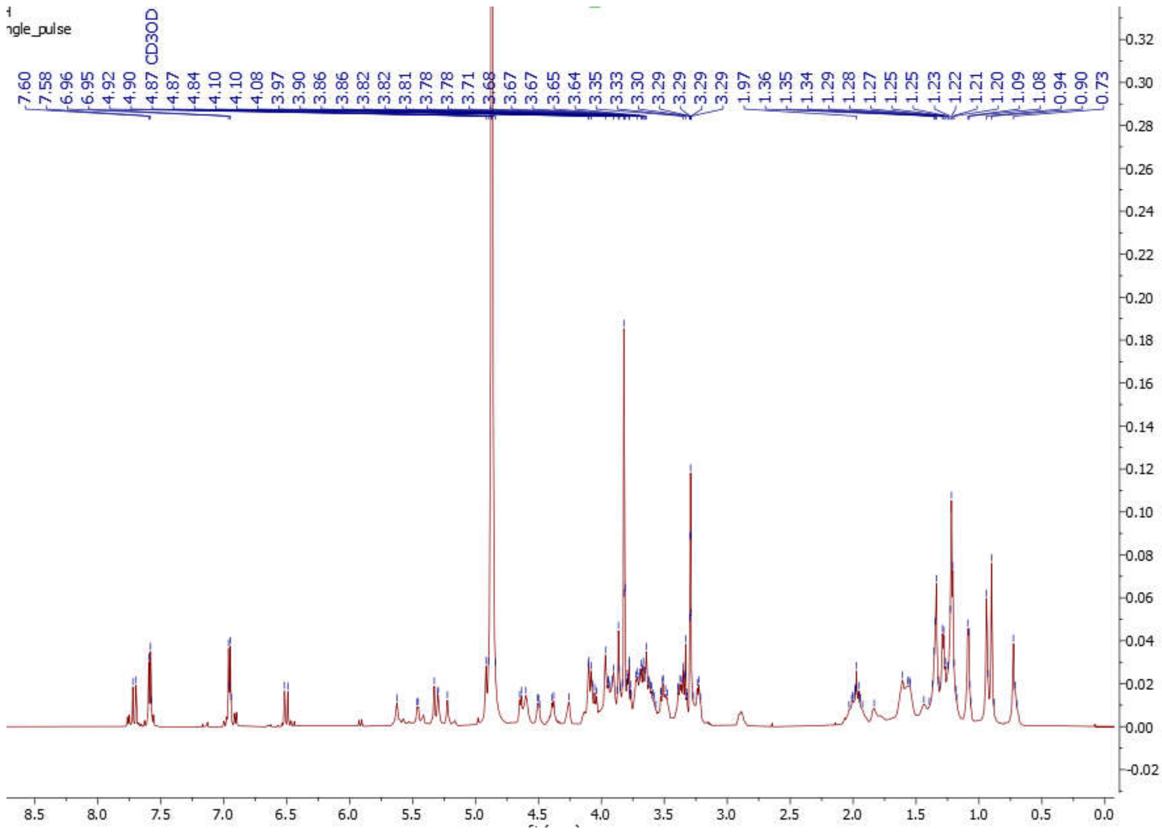

Figure S19. ^{1}H-NMR spectrum (MeOD, 600 MHz) of compound (**10**)

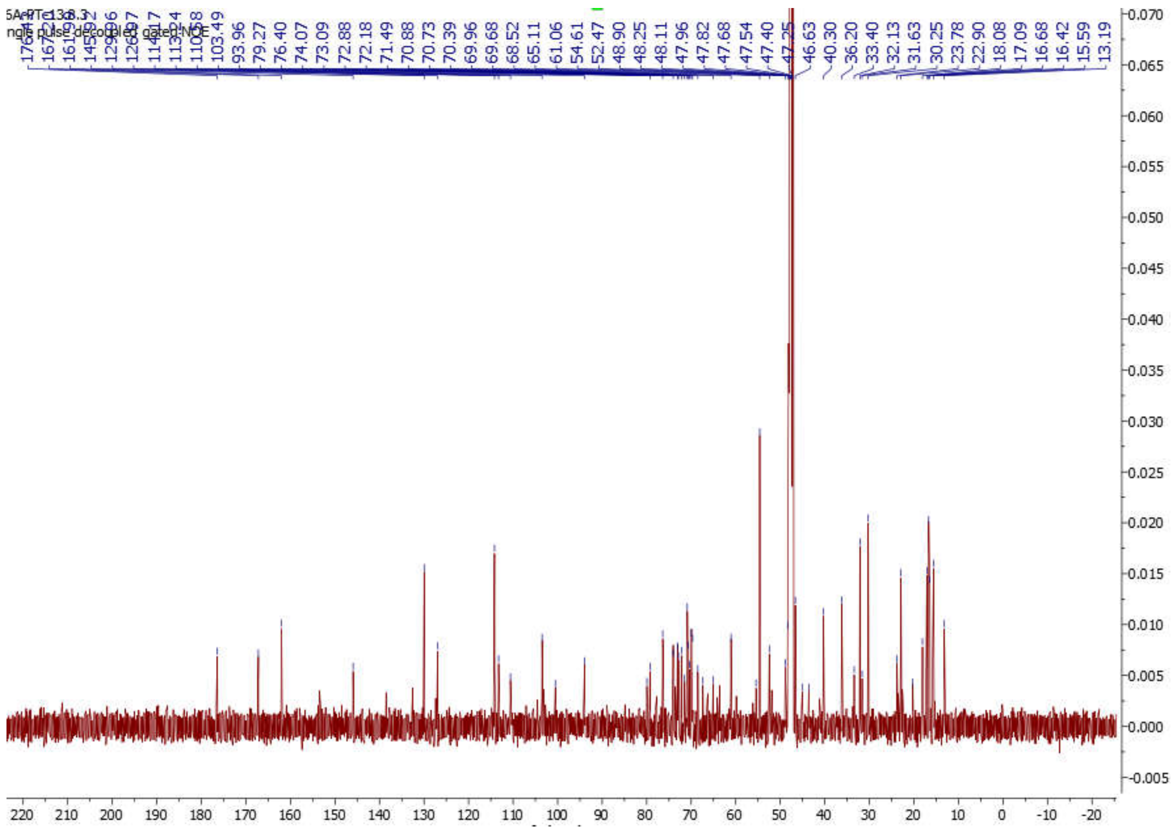

Figure S20. ^{13}C-NMR spectrum (MeOD, 150 MHz) of compound (**10**)

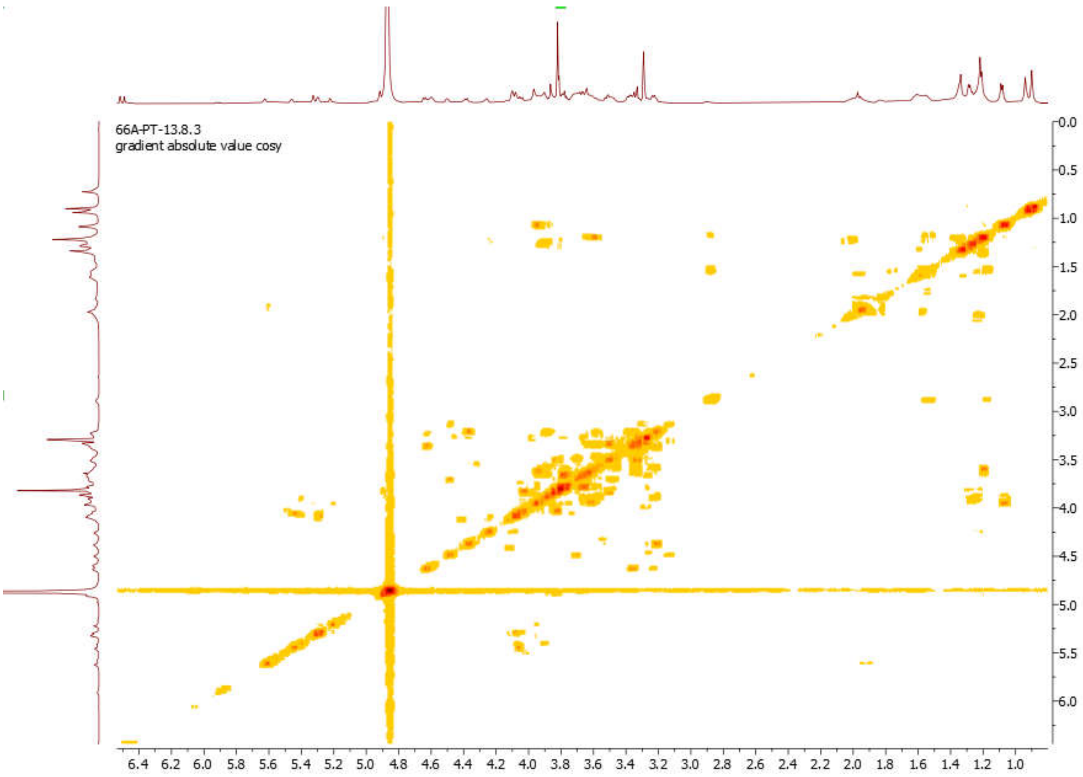

Figure S21. COSY spectrum (MeOD, 600 MHz) of compound (**10**)

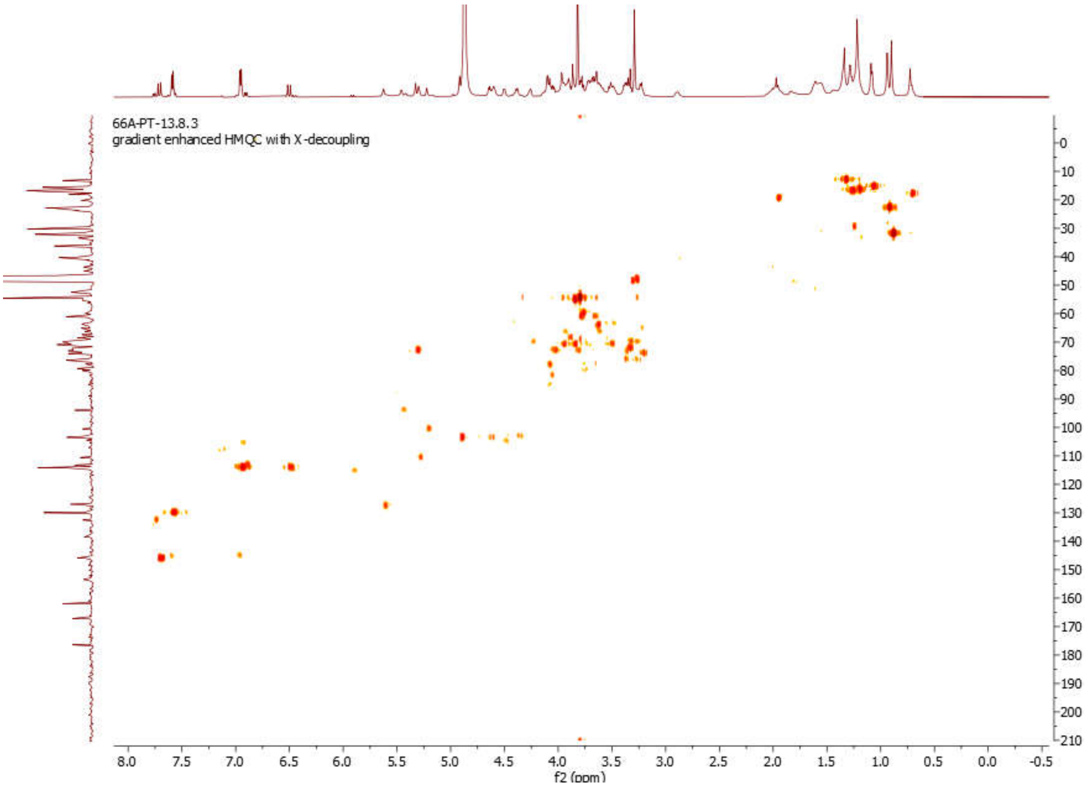

Figure S22. HMQC spectrum (MeOD, 600 MHz) of compound (**10**)

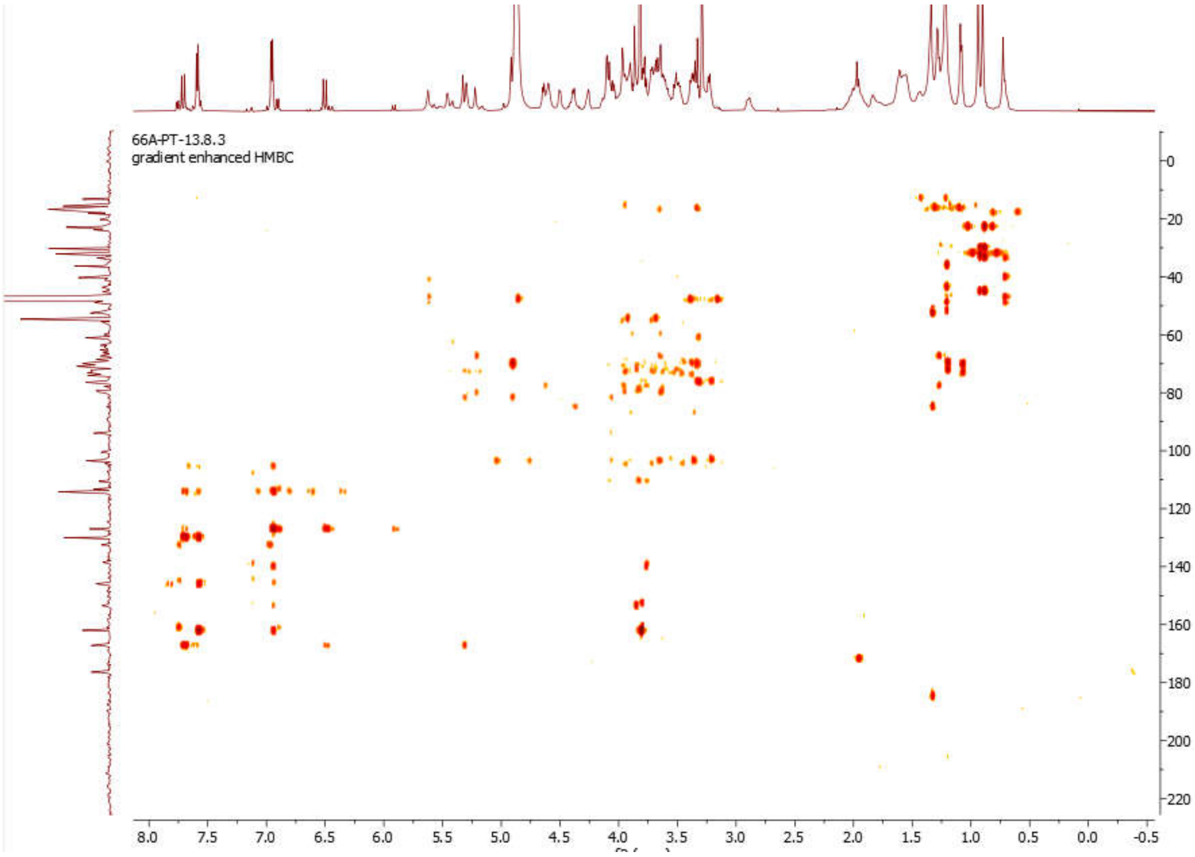

Figure S23. HMBC spectrum (MeOD, 600 MHz) of compound (**10**)

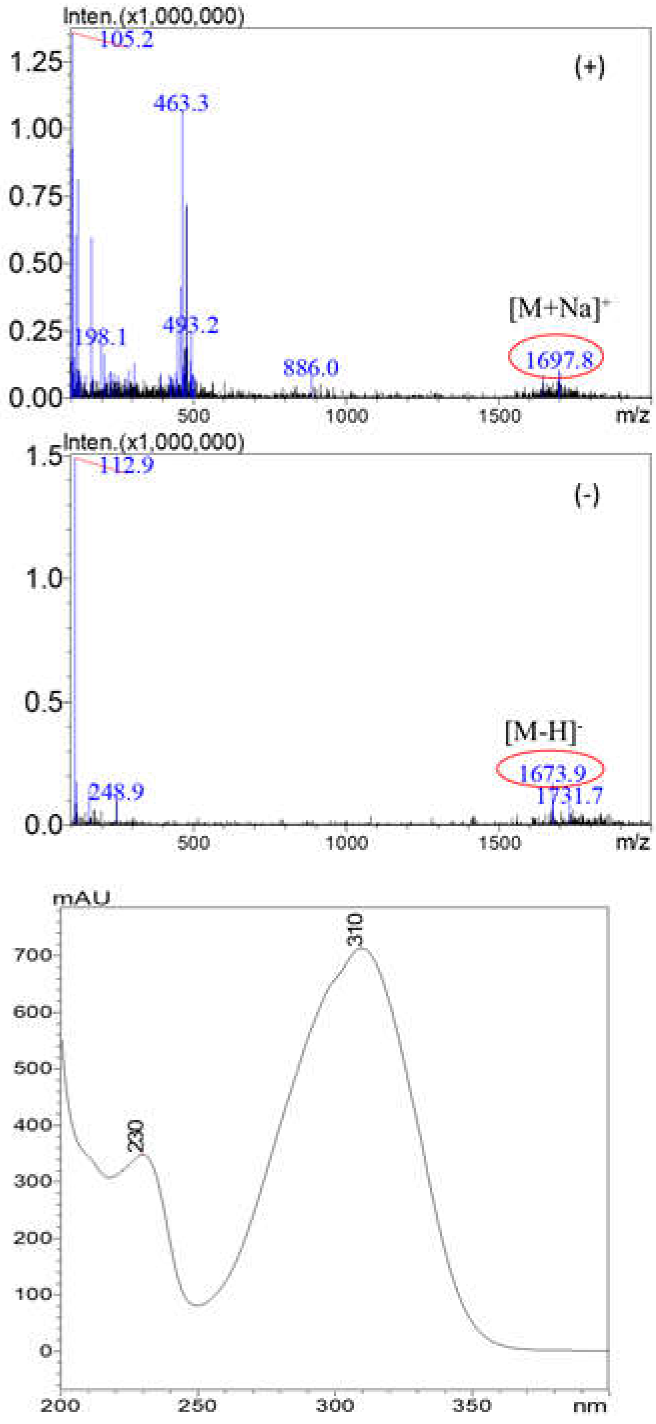

Figure S24. LC-MS spectrum of compound (**10**)

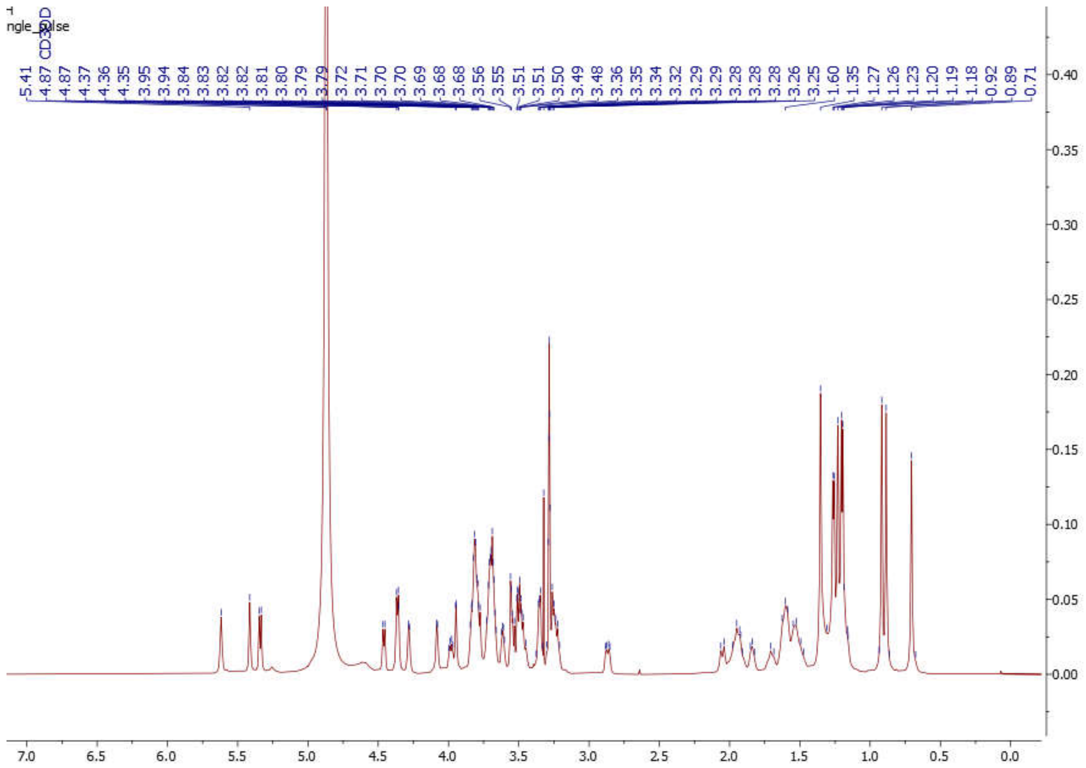

Figure S25. ^{1}H-NMR spectrum (MeOD, 600 MHz) of compound (**11**)

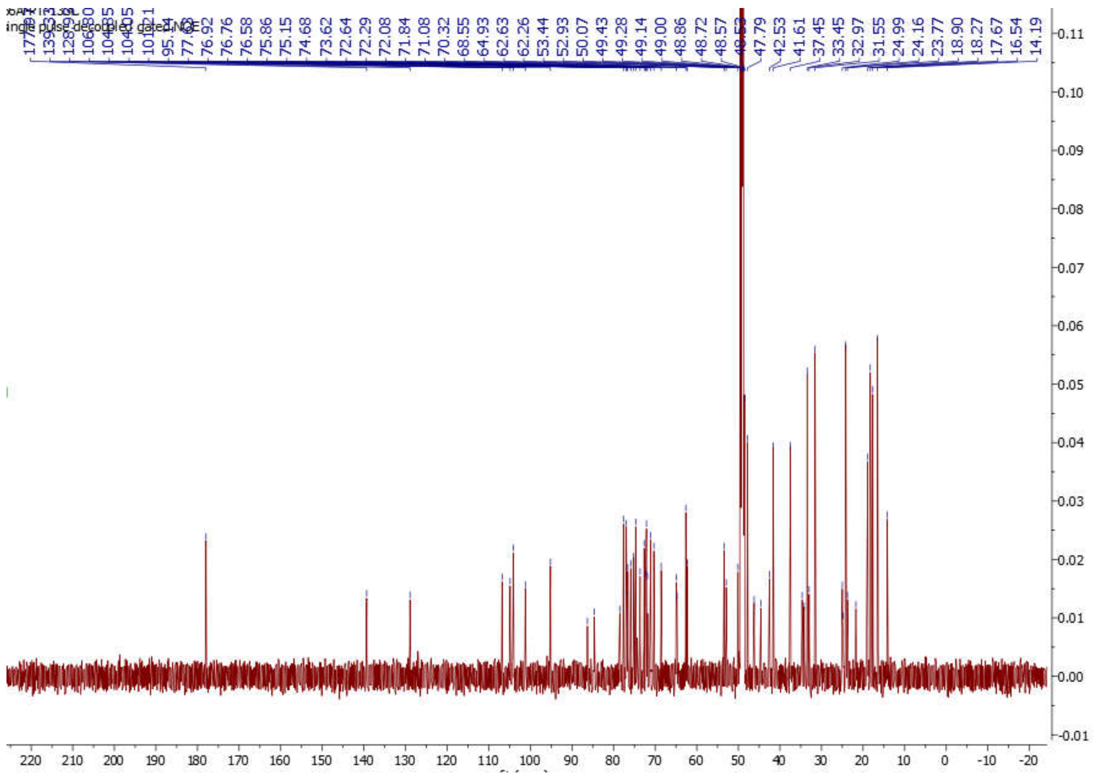

Figure S26. ^{13}C-NMR spectrum (MeOD, 150 MHz) of compound (**11**)

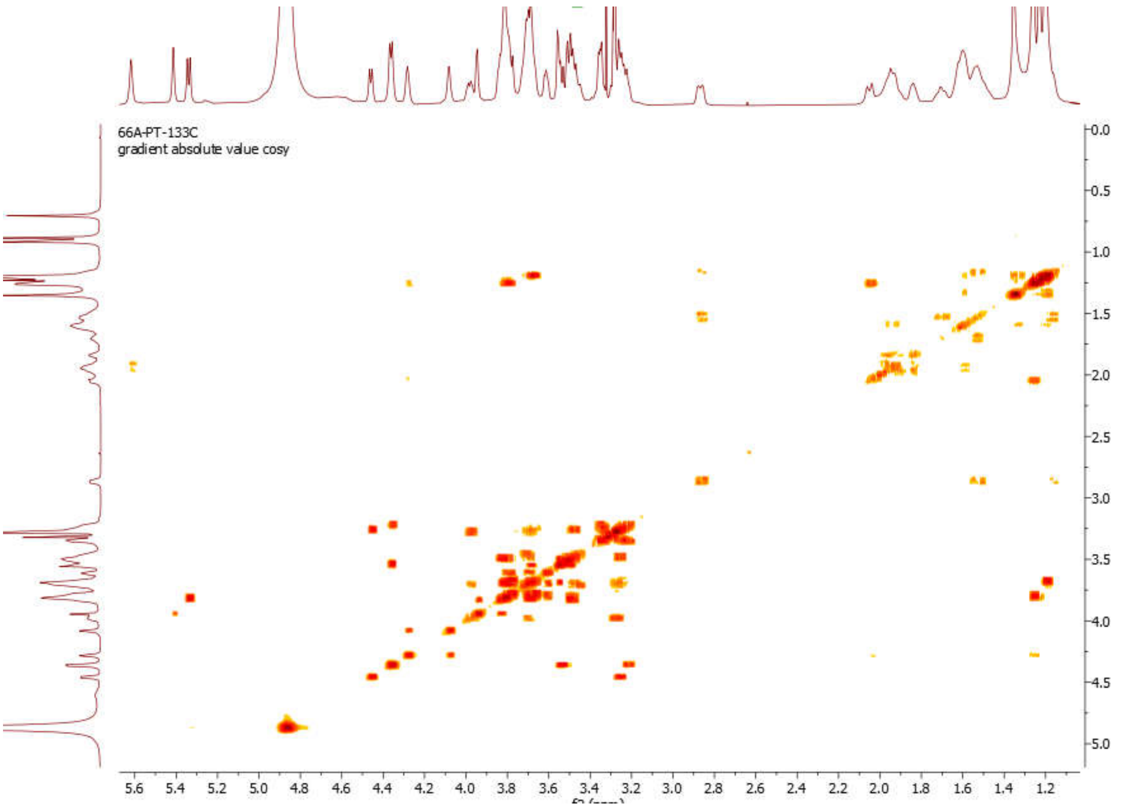

Figure S27. COSY spectrum (MeOD, 600 MHz) of compound (**11**)

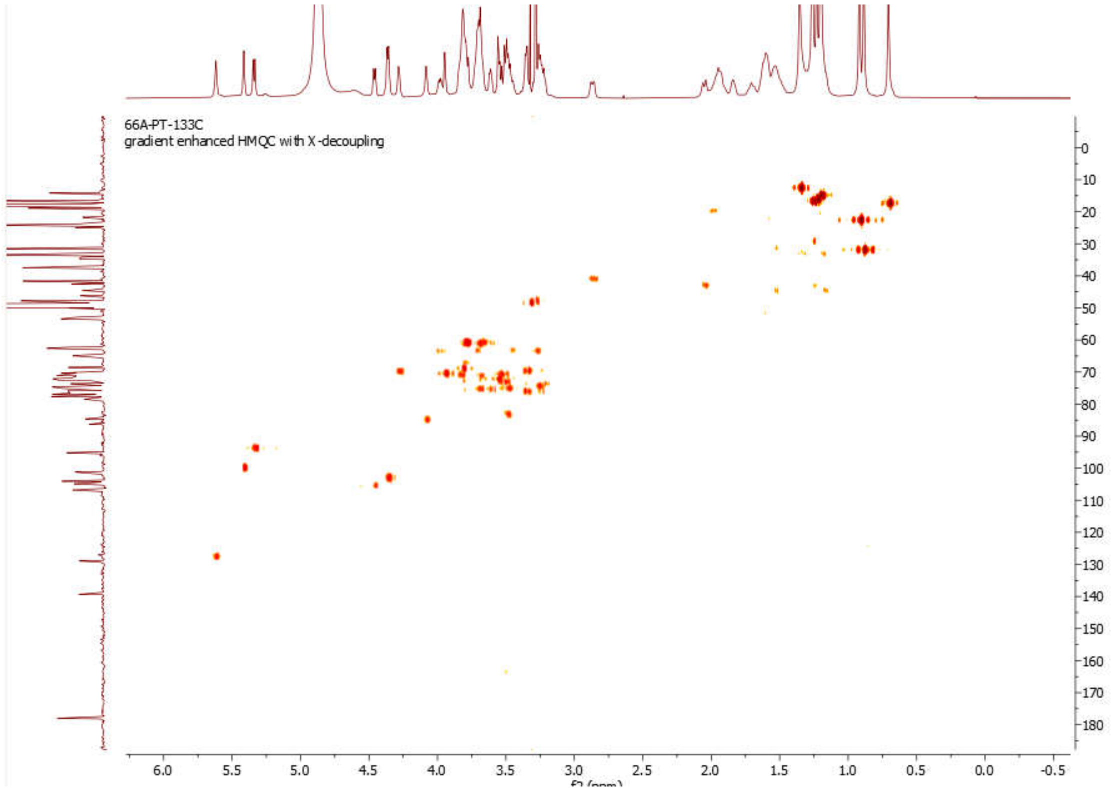

Figure S28. HMQC spectrum (MeOD, 600 MHz) of compound (**11**)

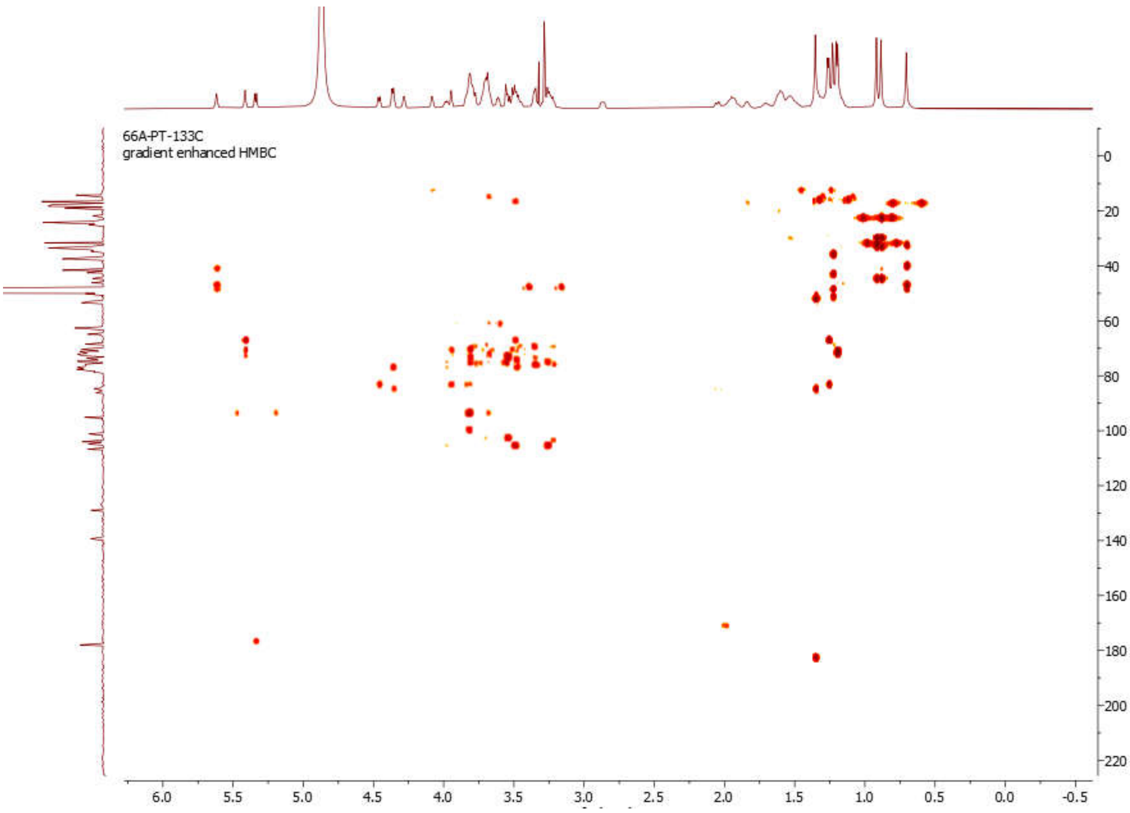

Figure S29. HMBC spectrum (MeOD, 600 MHz) of compound (**11**)

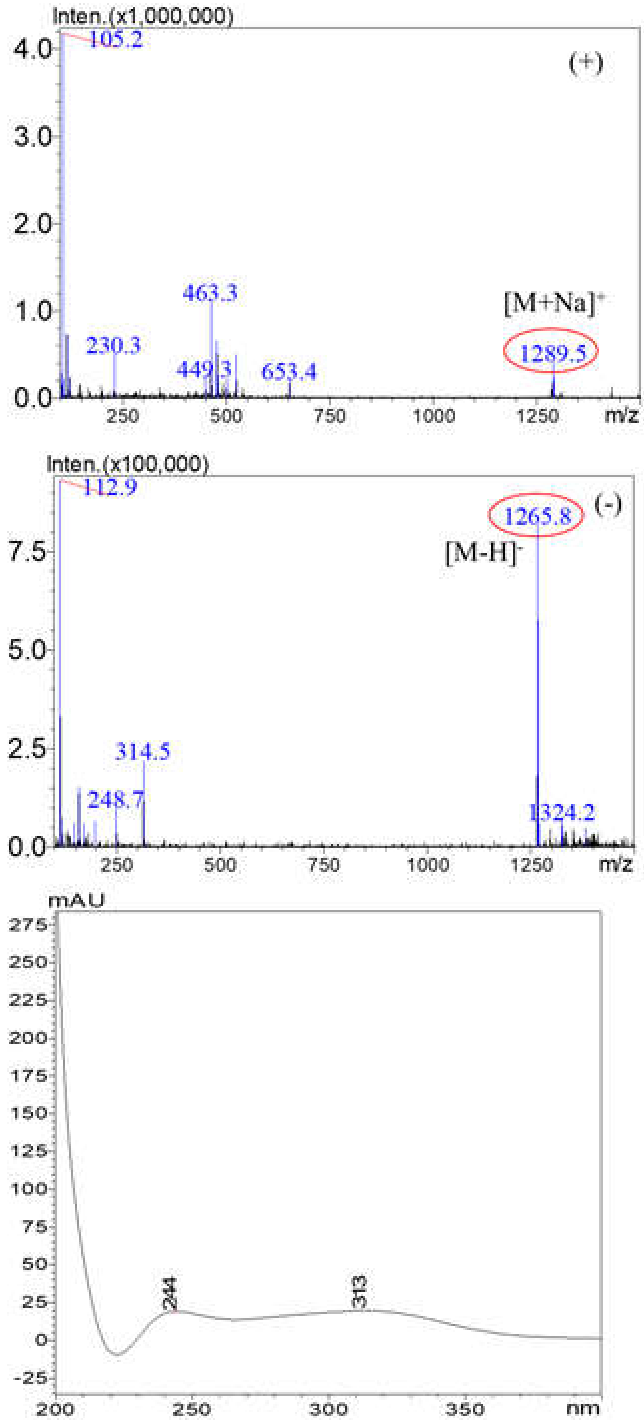

Figure S30. LC-MS spectrum of compound (**11**)

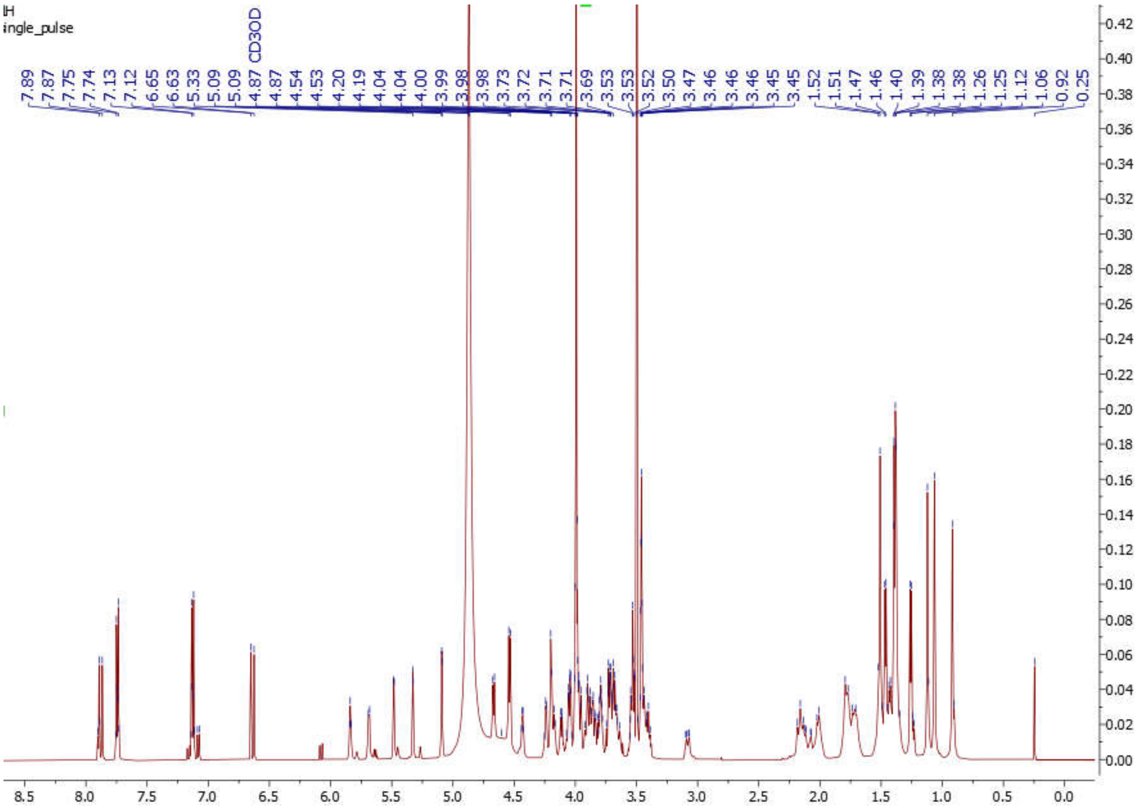

Figure S31. ^{1}H-NMR spectrum (MeOD, 600 MHz) of compound (**12**)

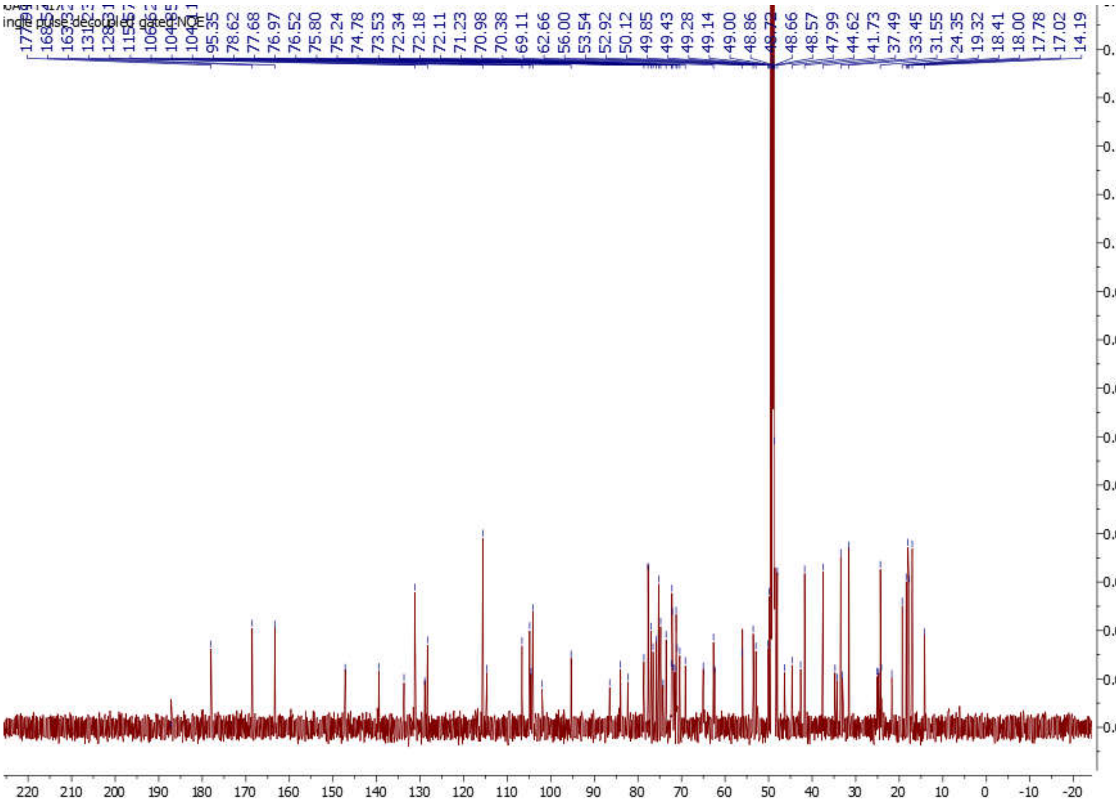

Figure S32. ^{13}C-NMR spectrum (MeOD, 150 MHz) of compound (**12**)

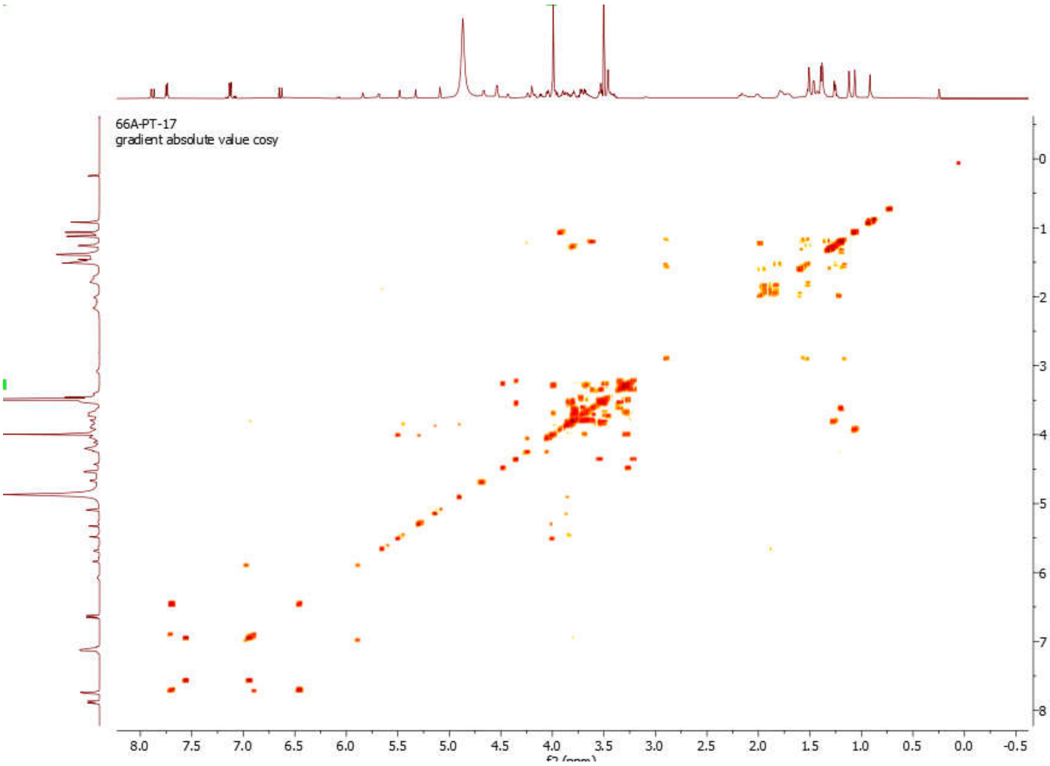

Figure S33. COSY spectrum (MeOD, 600 MHz) of compound (**12**)

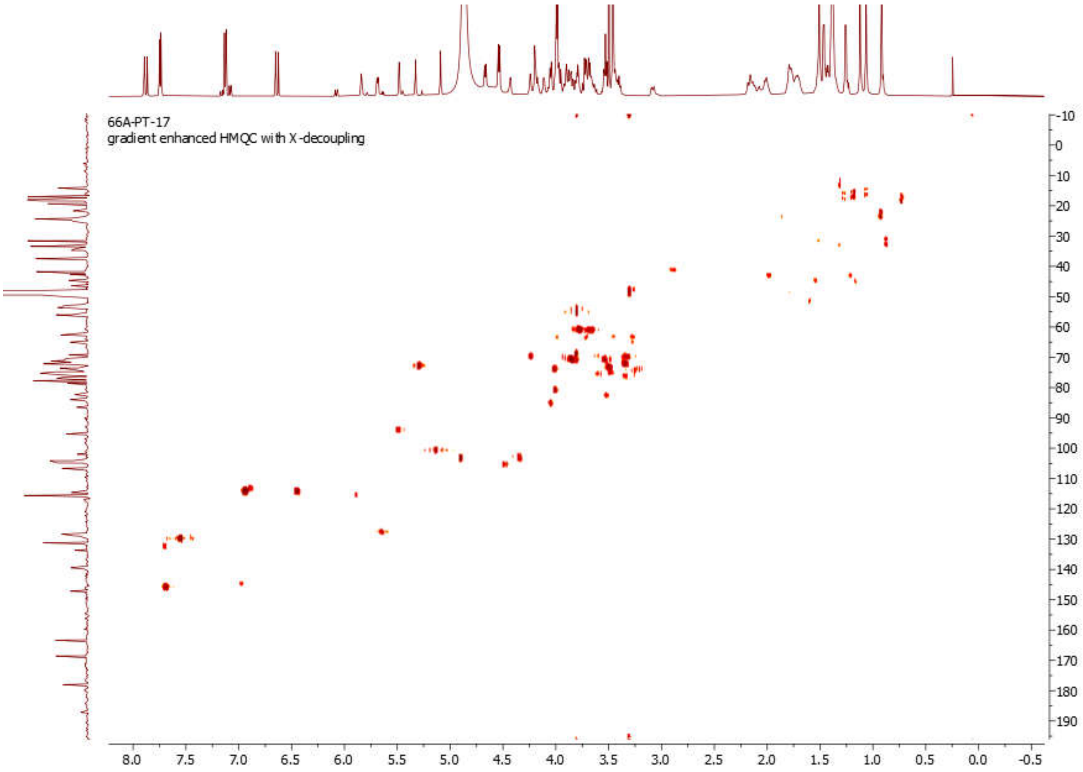

Figure S34. HMQC spectrum (MeOD, 600 MHz) of compound (**12**)

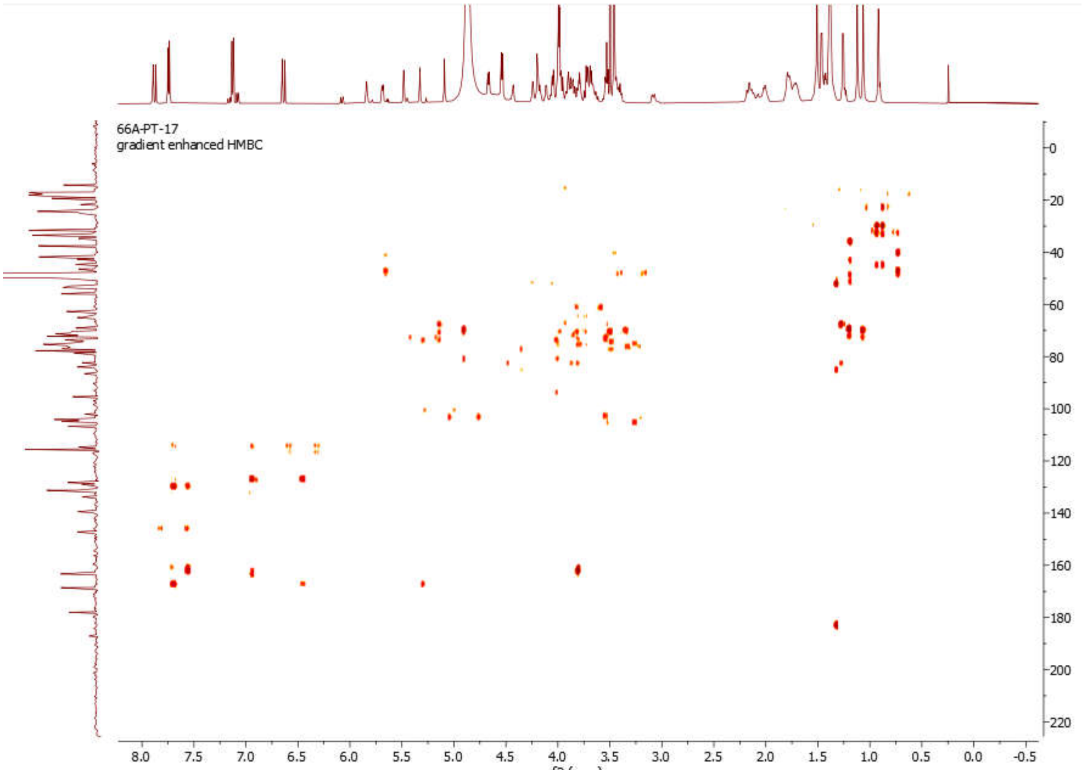

Figure S35. HMBC spectrum (MeOD, 600 MHz) of compound (**12**)

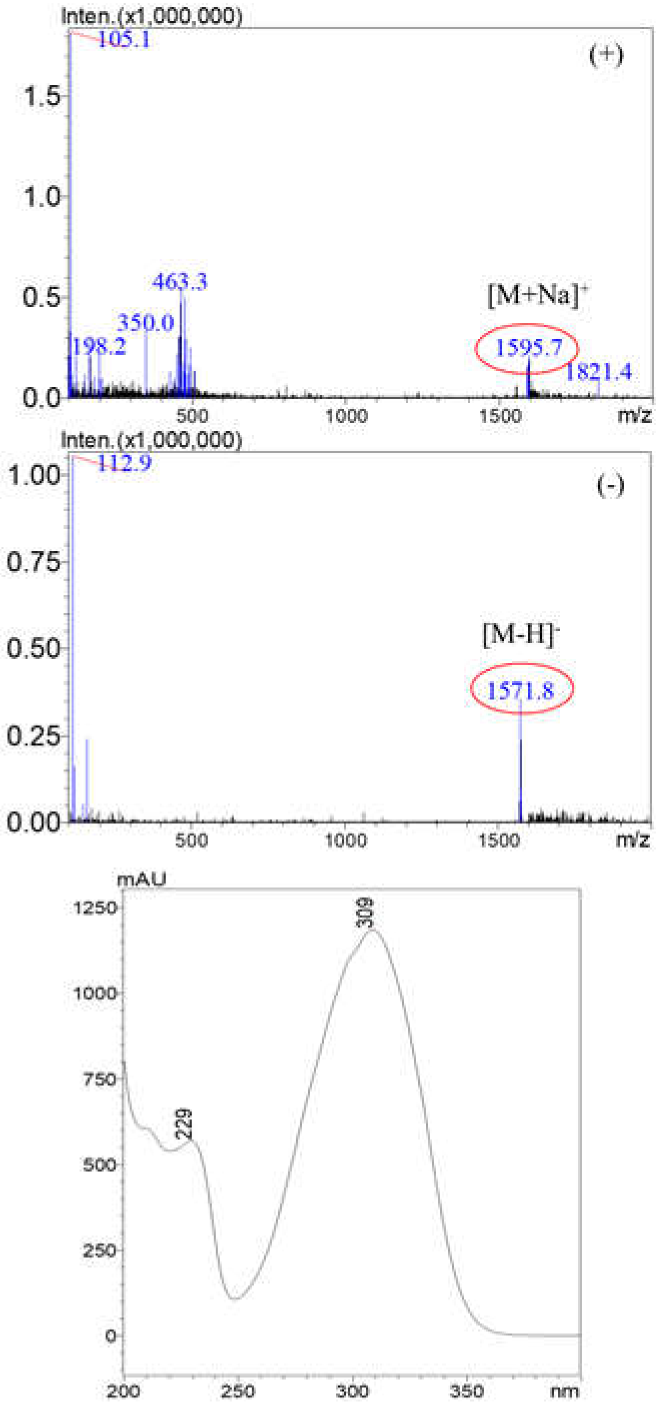

Figure S36. LC-MS spectrum of compound (**12**)

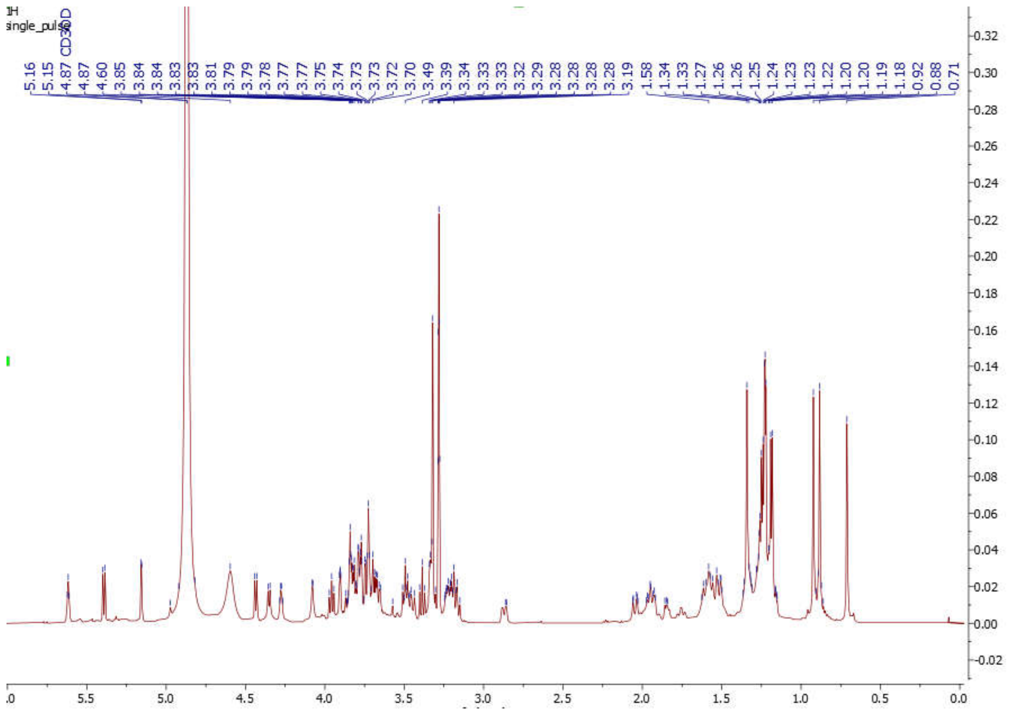

Figure S37. ^{1}H-NMR spectrum (MeOD, 600 MHz) of compound (**13**)

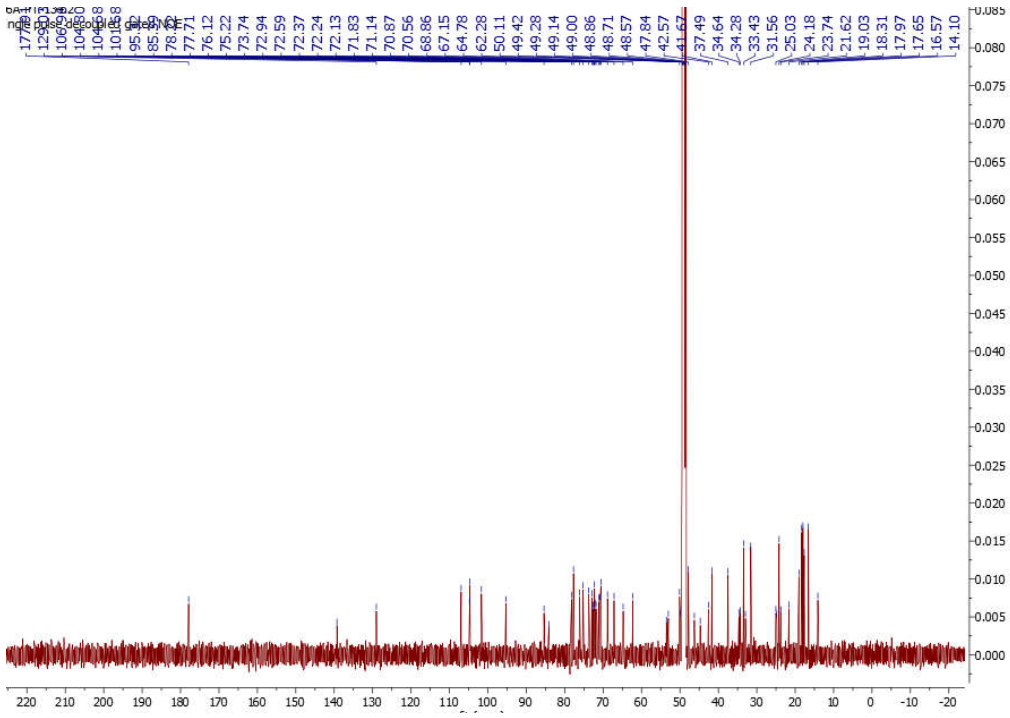

Figure S38. ^{13}C-NMR spectrum (MeOD, 150 MHz) of compound (**13**)

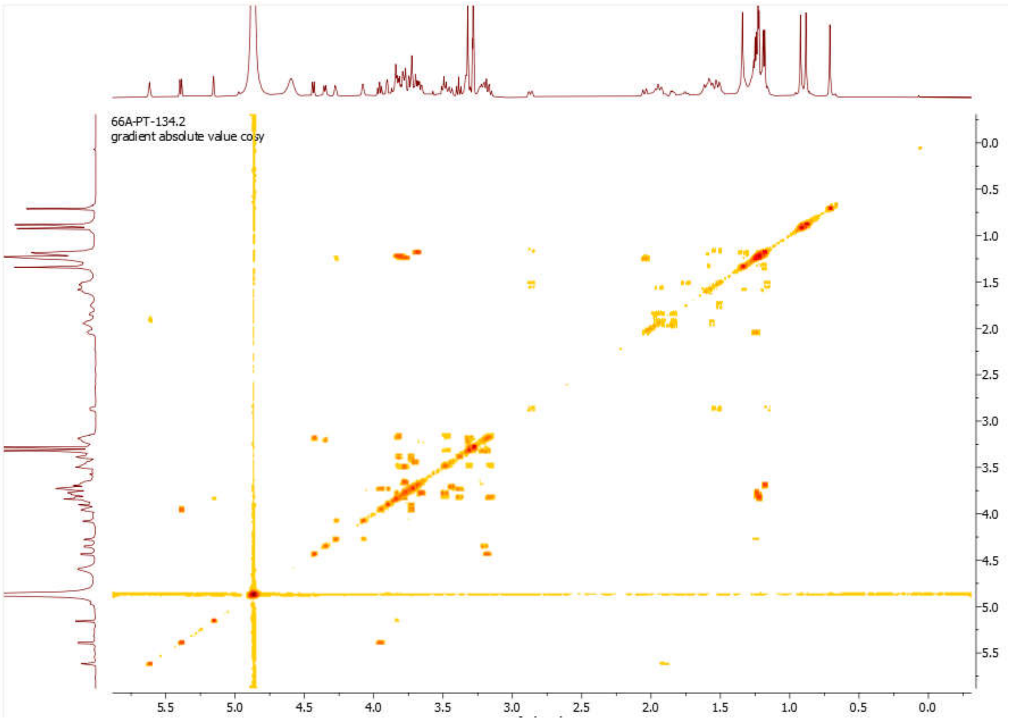

Figure S39. COSY spectrum (MeOD, 600 MHz) of compound (**13**)

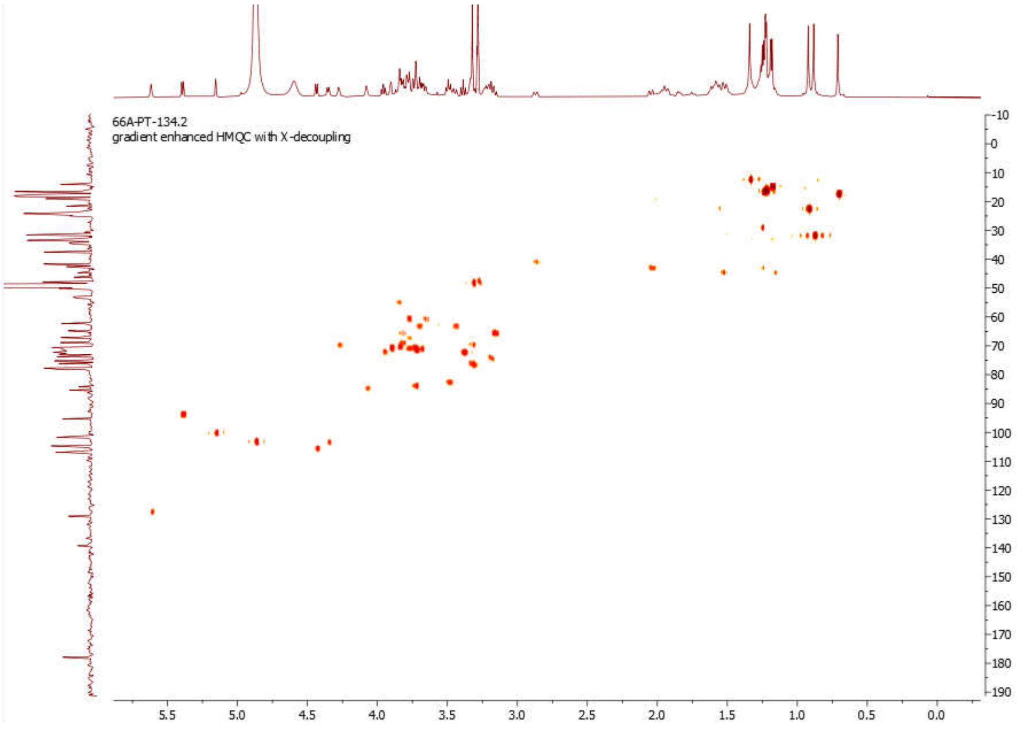

Figure S40. HMQC spectrum (MeOD, 600 MHz) of compound (**13**)

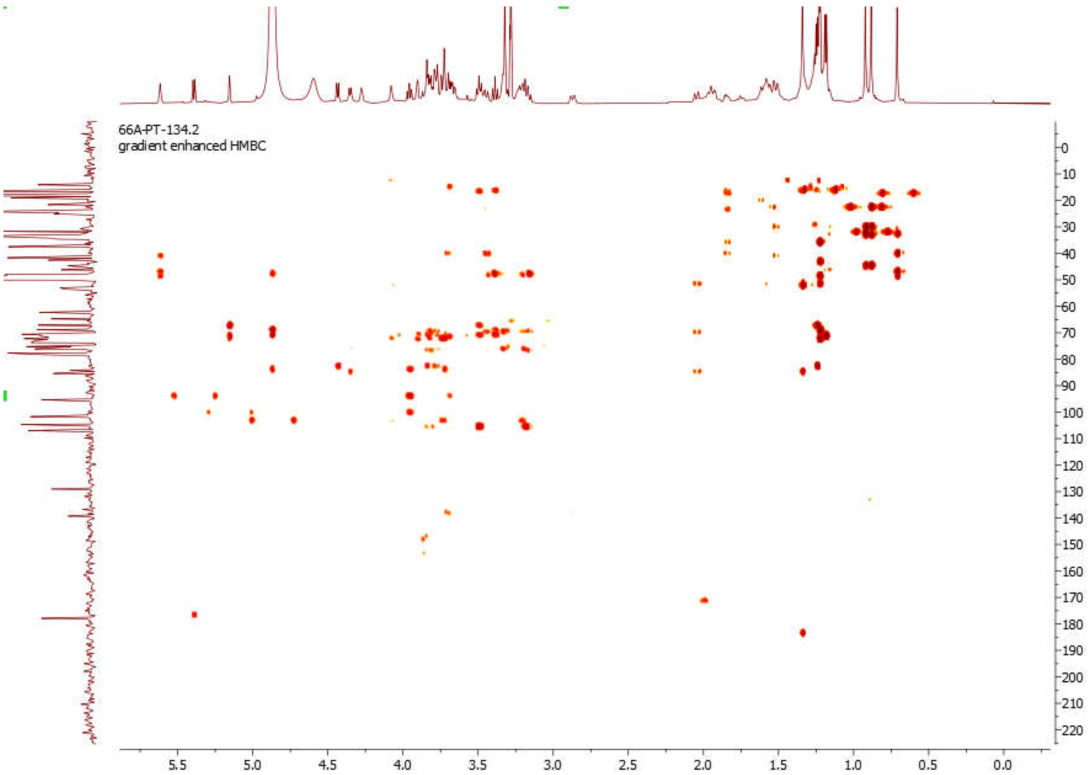

Figure S41. HMBC spectrum (MeOD, 600 MHz) of compound (**13**)

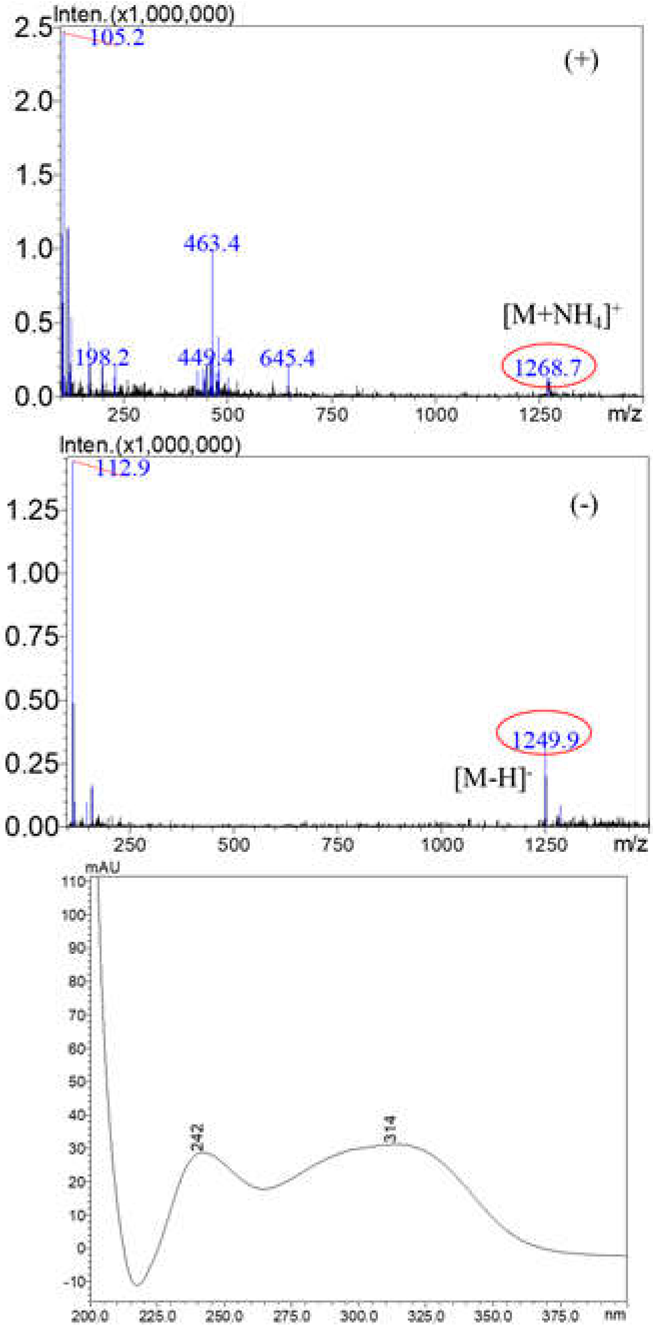

Figure S42. LC-MS spectrum of compound (**13**)

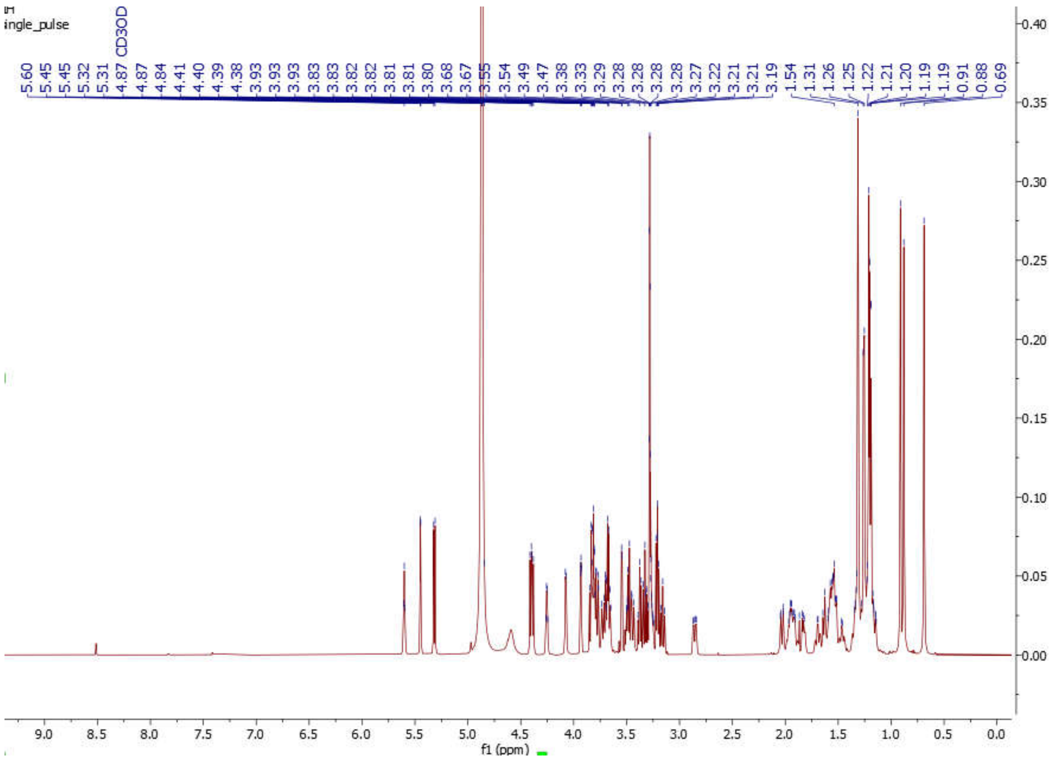

Figure S43. ^{1}H-NMR spectrum (MeOD, 600 MHz) of compound (**14**)

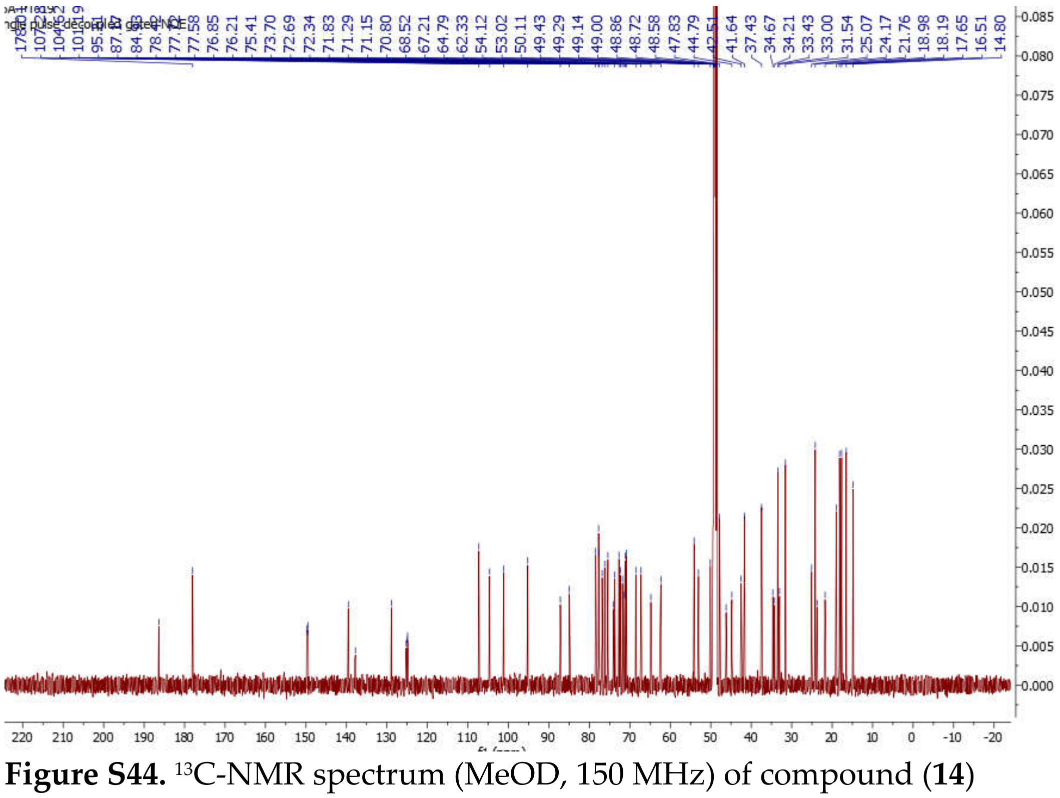

Figure S44. ^{13}C-NMR spectrum (MeOD, 150 MHz) of compound (**14**)

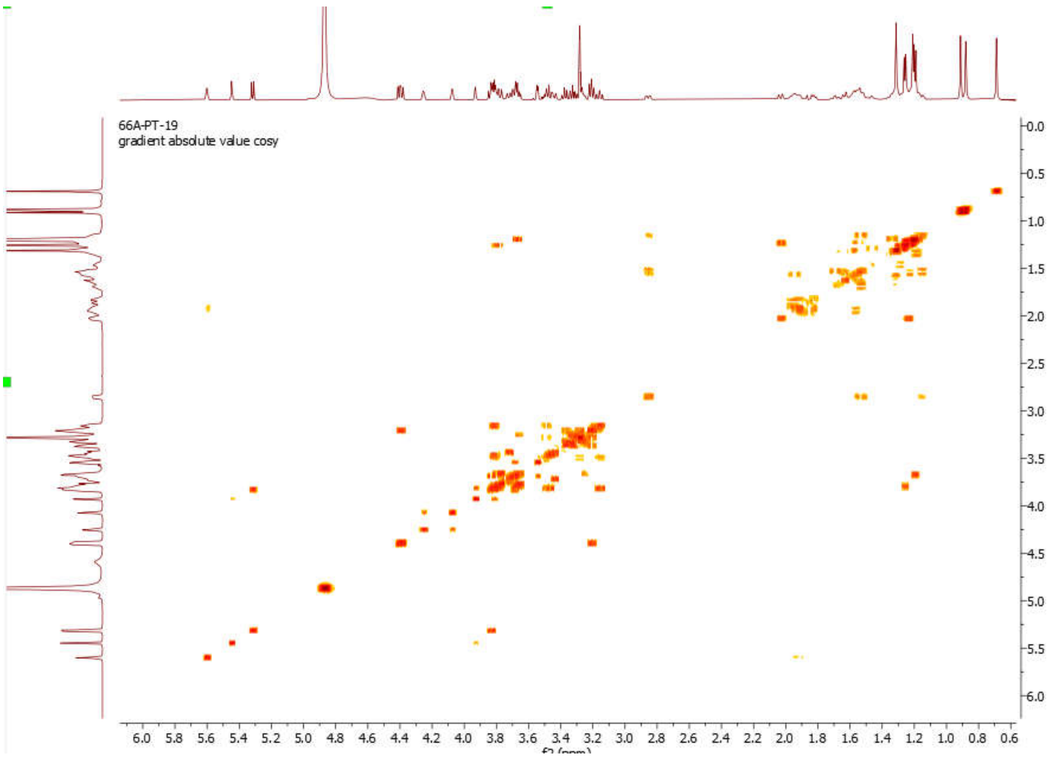

Figure S45. COSY spectrum (MeOD, 600 MHz) of compound (**14**)

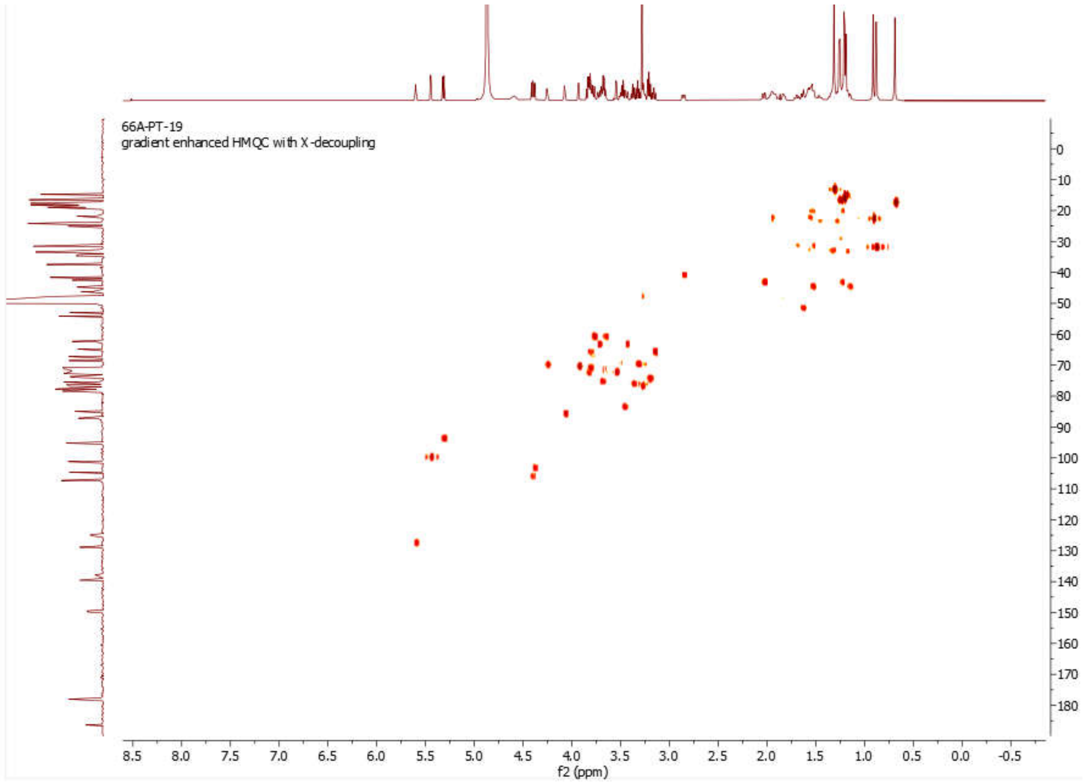

Figure S46. HMQC spectrum (MeOD, 600 MHz) of compound (**14**)

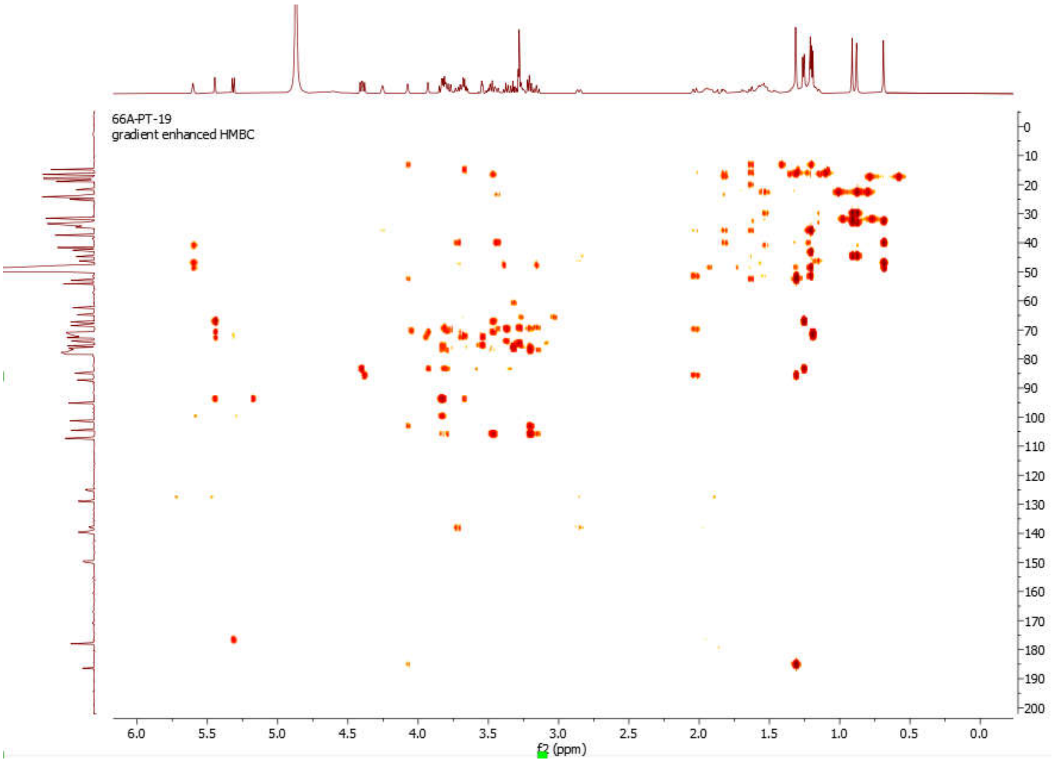

Figure S47. HMBC spectrum (MeOD, 600 MHz) of compound (**14**)

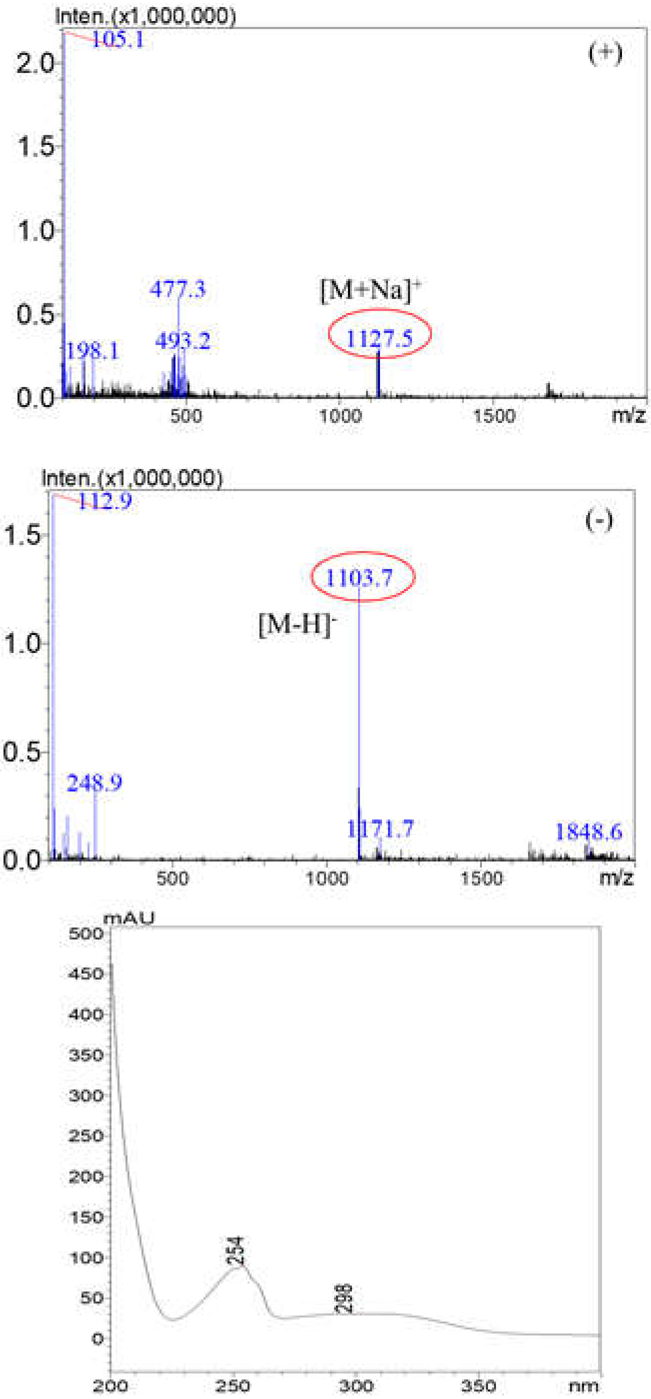

Figure S48. LC-MS spectrum of compound (**14**)

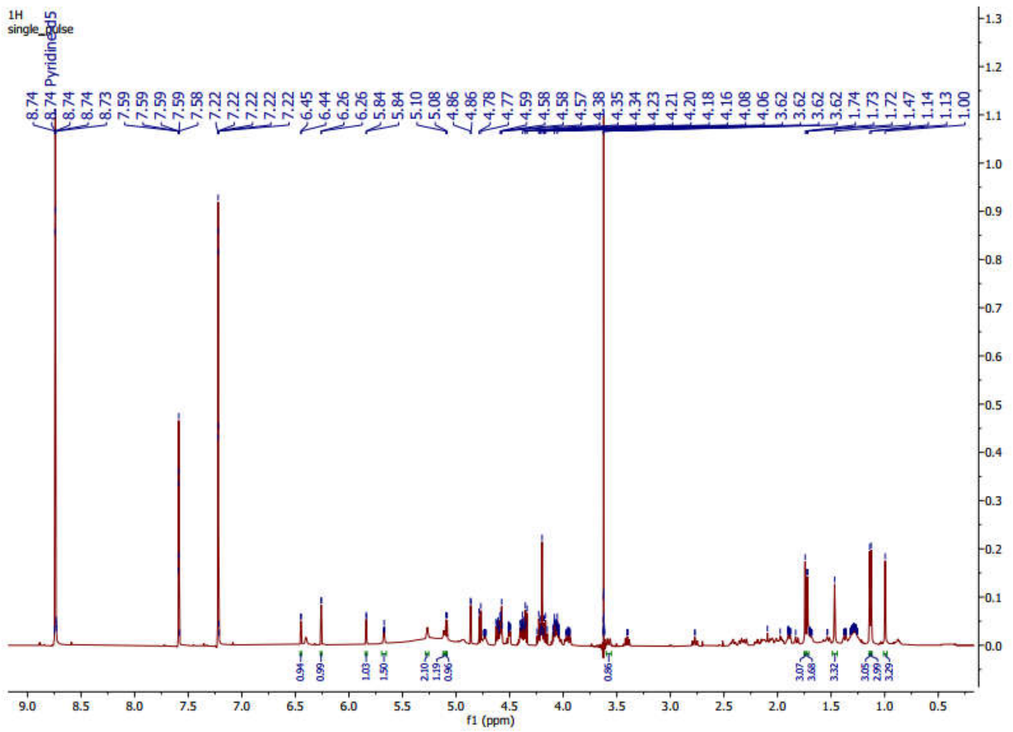

Figure S49. ^{1}H-NMR spectrum (C_5D_5N, 600 MHz) of compound (**15**)

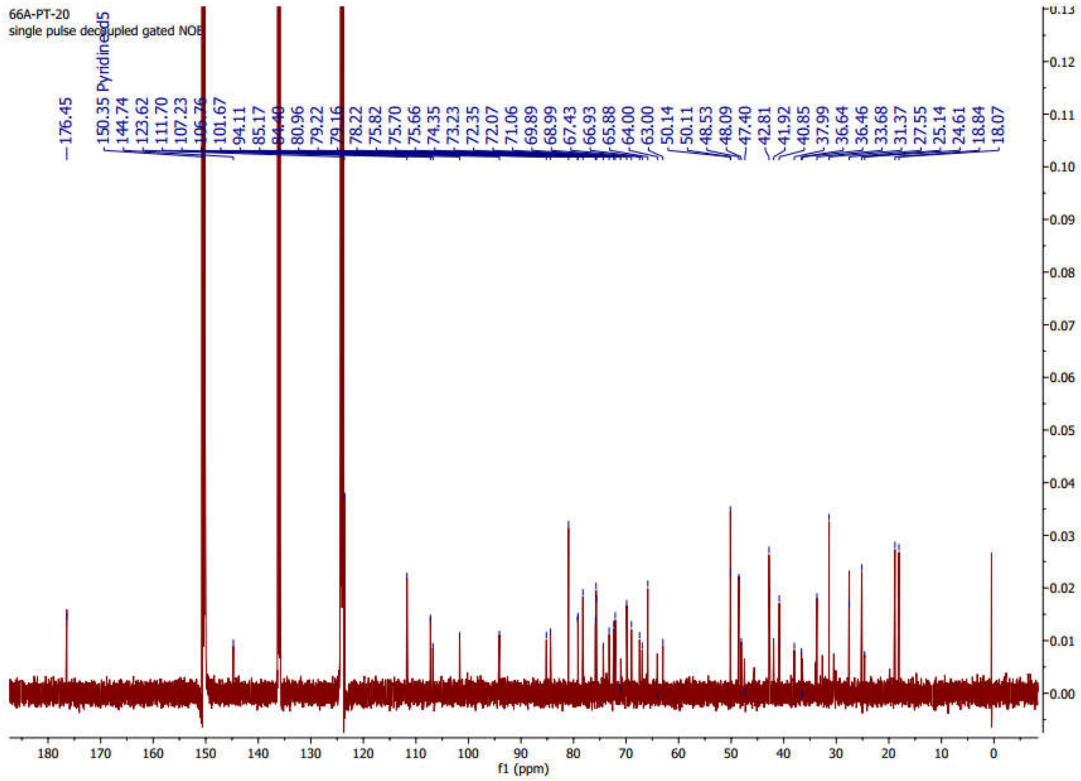

Figure S50. 13C-NMR spectrum (C_5D_5N, 150 MHz) of compound (**15**)

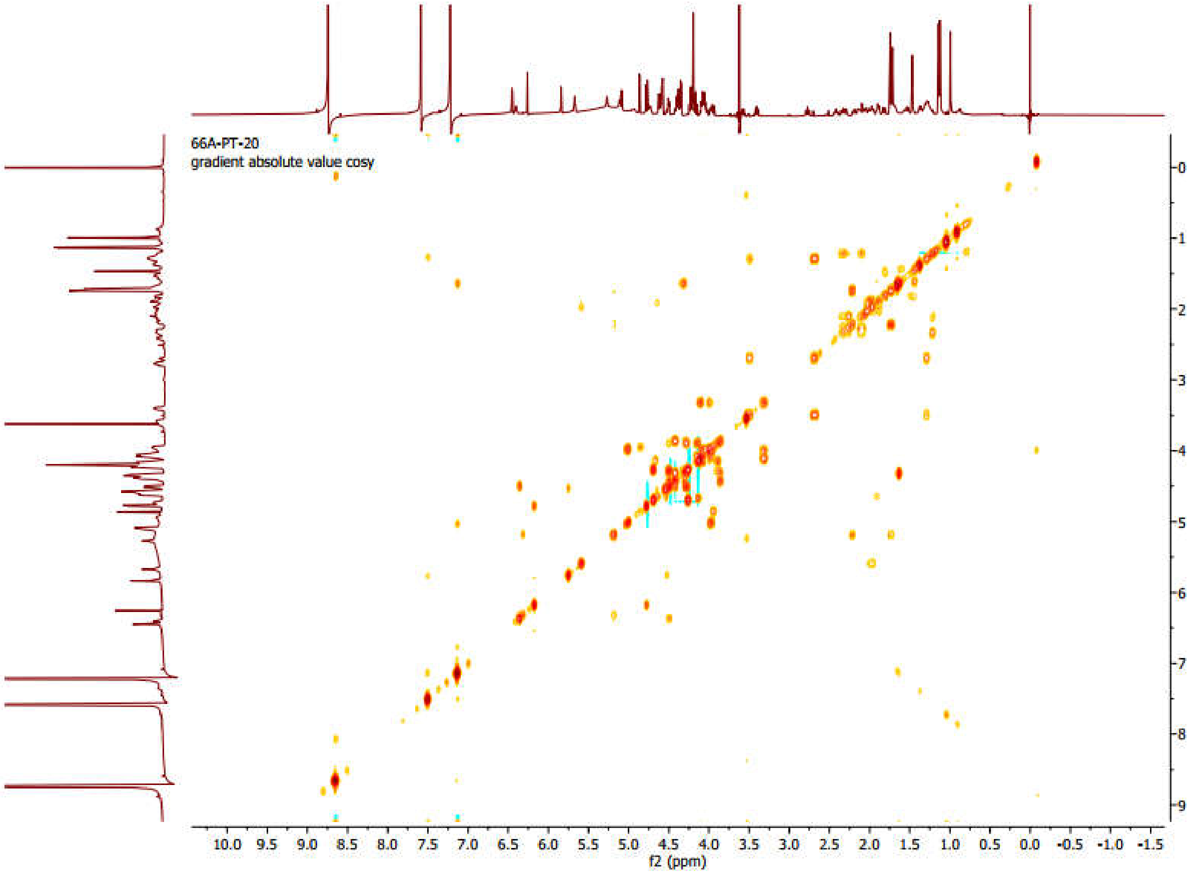

Figure S51. COSY spectrum (C_5D_5N, 600 MHz) of compound (**15**)

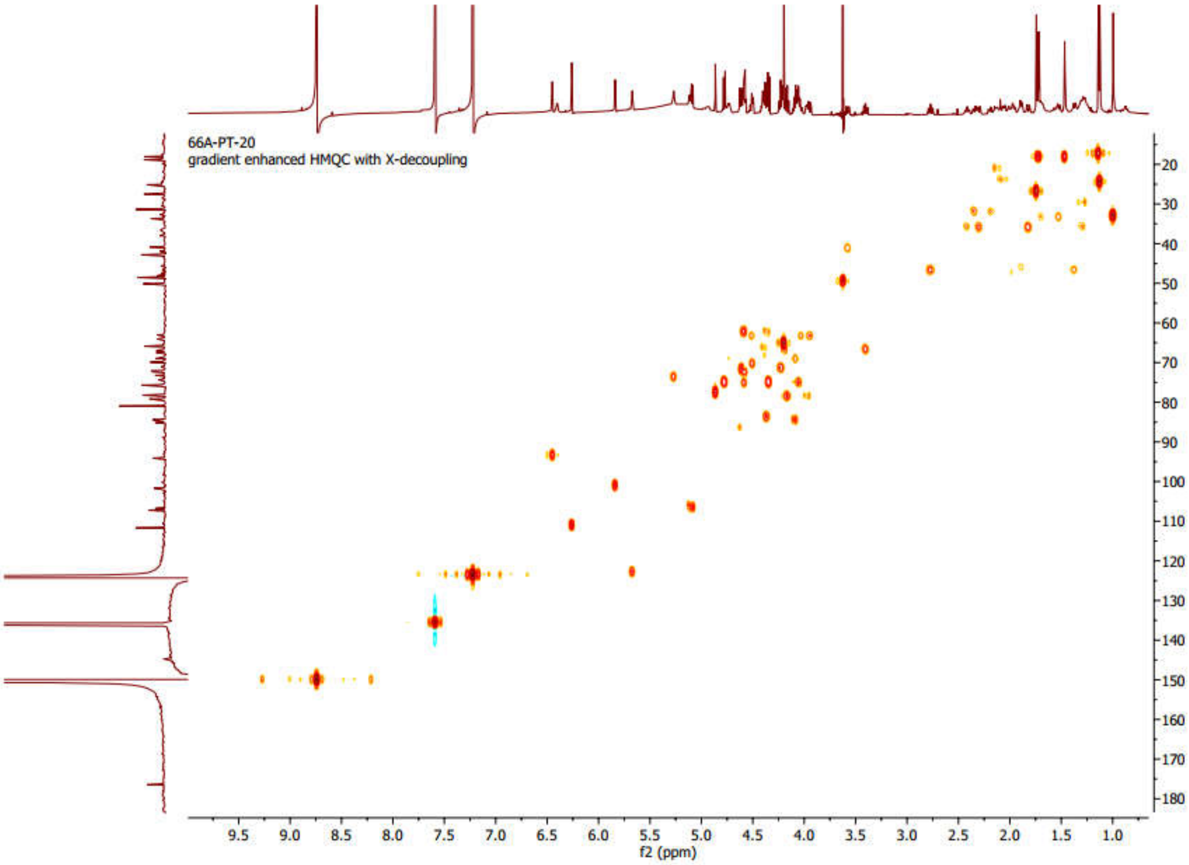

Figure S52. HMQC spectrum (C_5D_5N, 600 MHz) of compound (**15**)

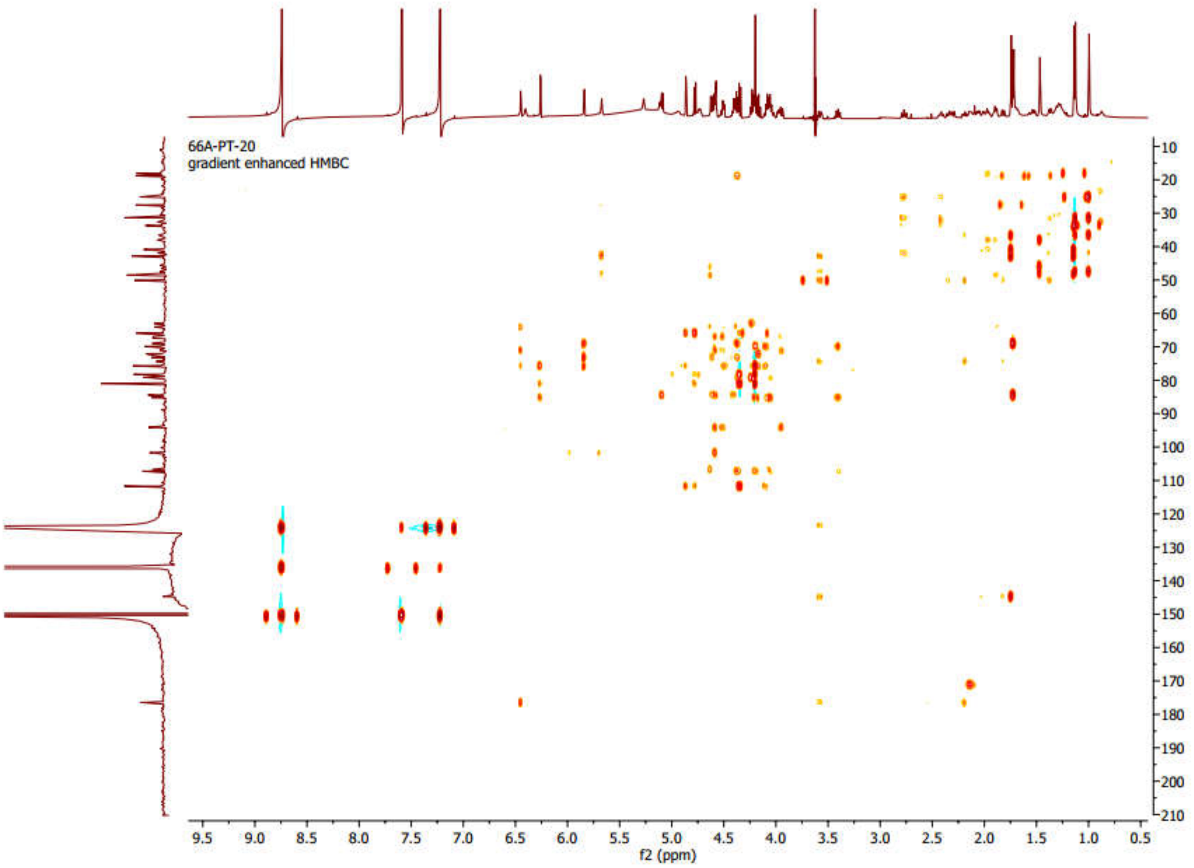

Figure S53. HMBC spectrum (C_5D_5N, 600 MHz) of compound (**15**)

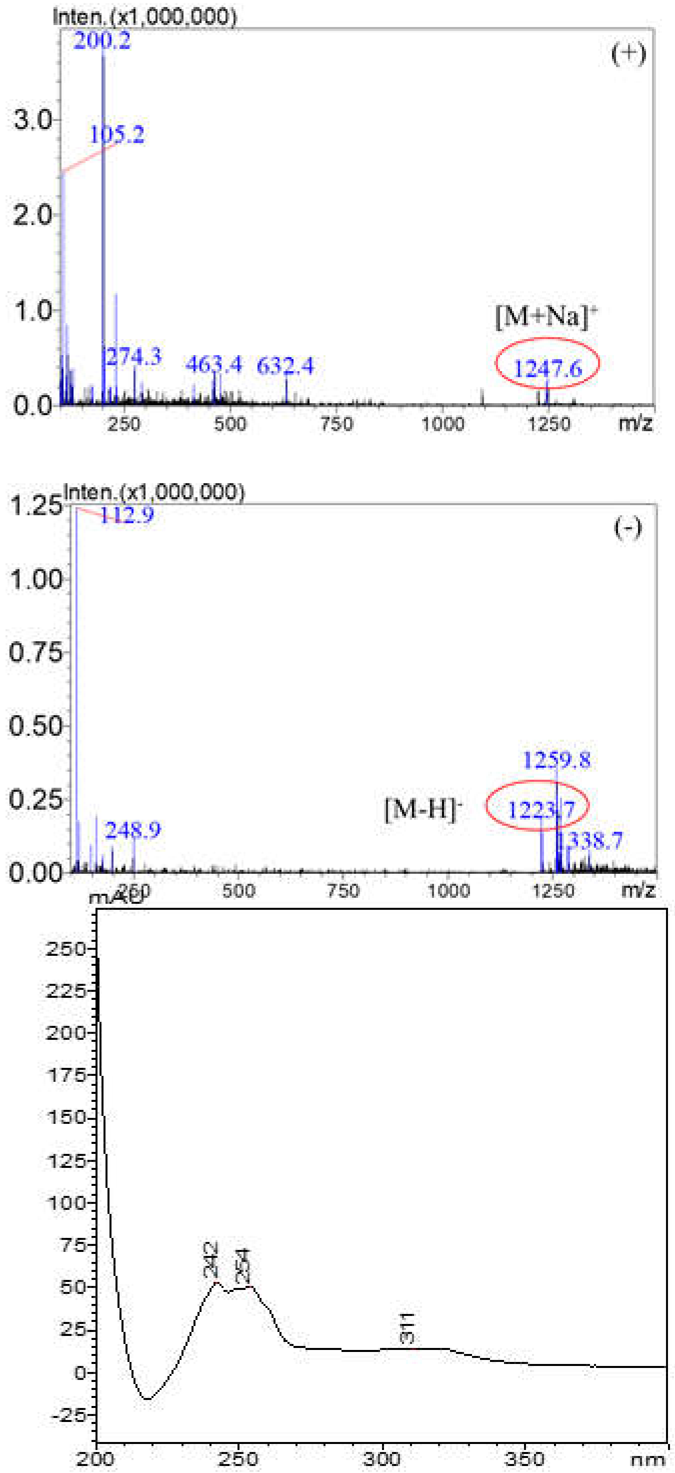

Figure S54. LC-MS spectrum of compound (**15**)
